# Development of multifunctional Overhauser-enhanced magnetic resonance imaging for concurrent *in vivo* mapping of tumor interstitial oxygenation, acidosis and inorganic phosphate concentration

**DOI:** 10.1038/s41598-019-48524-3

**Published:** 2019-08-20

**Authors:** Artem A. Gorodetskii, Timothy D. Eubank, Benoit Driesschaert, Martin Poncelet, Emily Ellis, Valery V. Khramtsov, Andrey A. Bobko

**Affiliations:** 1 In Vivo Multifunctional Magnetic Resonance center, Robert C. Byrd Health Sciences Center, West Virginia University, Morgantown, WV 26506 USA; 2 Department of Biochemistry, West Virginia University School of Medicine, Morgantown, WV 26506 USA; 3 N.N. Voroztsov Novosibirsk Institute of Organic Chemistry SB RAS, Novosibirsk, 630090 Russia; 4 Novosibirsk State University, Novosibirsk, 630090 Russia; 5 Department of Microbiology, Immunology & Cell Biology, West Virginia University School of Medicine, Morgantown, WV 26506 USA; 6 Department of Pharmaceutical Sciences, West Virginia University, School of Pharmacy, Morgantown, WV 26506 USA; 7 West Virginia University Cancer Institute, Morgantown, WV 26506 USA

**Keywords:** Cancer microenvironment, Breast cancer, Cancer imaging

## Abstract

Tumor oxygenation (*p*O_2_), acidosis (pH) and interstitial inorganic phosphate concentration (Pi) are important parameters of the malignant behavior of cancer. A noninvasive procedure that enables visualization of these parameters may provide unique information about mechanisms of tumor pathophysiology and provide clues to new treatment targets. In this research, we present a multiparametric imaging method allowing for concurrent mapping of pH, spin probe concentration, *p*O_2_, and Pi using a single contrast agent and Overhauser-enhanced magnetic resonance imaging technique. The developed approach was applied to concurrent multifunctional imaging in phantom samples and *in vivo* in a mouse model of breast cancer. Tumor tissues showed higher heterogeneity of the distributions of the parameters compared with normal mammary gland and demonstrated the areas of significant acidosis, hypoxia, and elevated Pi content.

## Introduction

Solid tumors develop a tissue microenvironment profile significantly different from parent healthy tissue. Tissue oxygenation (*p*O_2_), interstitial pH, and inorganic phosphate (Pi) levels are important markers of tumorigenic activity^1–5^. Magnetic resonance techniques are widely used to study cancer in both preclinical animals model and clinical set-ups. Magnetic resonance imaging (MRI) is one of the most successful techniques for anatomical tissue characterization, including tumor neoplasm detection. Moreover, MRI can be applied for measurements and mapping of oxygen^6–9^, pH^10–12^, and inorganic phosphate^13,14^ using both endogenous and exogenous molecular probes. In contrast to MRI, electron paramagnetic resonance (EPR) techniques are unsuitable for anatomical tissue characterization because of strict reliance on exogenous probes. However, EPR techniques have found multiple applications in various analyte detection, including *p*O_2_ ^15–18^, pH^18–21^, and Pi^4^. The major advantage of EPR techniques over MRI is multifunctionality of tissue parameter detection^20–24^. The double resonance imaging technique, Overhauser MRI (OMRI), combines advantages of both approaches allowing for concurrent anatomical tissue imaging and multifunctional mapping of tissue physiological parameters^25–29^. In OMRI, saturation of spin probe EPR transition by powerful radiofrequency irradiation results in polarization transfer from electron to nuclear spin (water protons in ^1^H MRI modality) transition levels and corresponding significant enhancement of MRI signal intensity. The variation of EPR saturation power or EPR frequency value allows for measurement of different analytes in solution (e.g., oxygen, pH et cet.)^25^.

Most OMRI experiments monitor concentrations of spin probes (nitroxide probes, redox-related studies^30–34^) or oxygenation level (trityl probes^26,34–37^). Several attempts were made to use the OMRI technique to map the acidity of tissues and solutions using both nitroxide^20,38^ and trityl^39^ probes. Here we present the development of a multifunctional OMRI technique using a monophosphonated trityl probe for concurrent mapping of several functional parameters, namely *p*O_2_, pH, Pi, and spin probe concentration.

## Results

### Concurrent pH, *p*O_2_, and Pi and contrast agent concentration measurements

**Figure 1:**
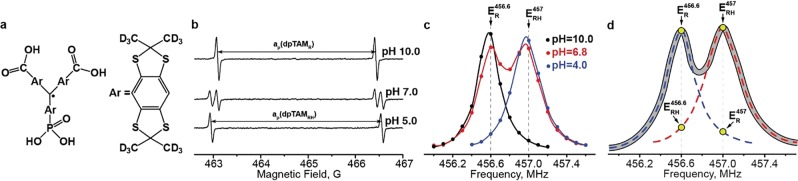
(**a**) Chemical structure of deuterated phosphonated trityl radical (dpTAM). (**b**) EPR spectra of dpTAM radical at different pH; a_p_(dpTAM_R_) and a_p_(dpTAM_RH_) are phosphorus hyperfine splitting constants for deprotonated and protonated forms, correspondingly. (**c**) The high frequency component of DNP spectra of 1 mM dpTAM solution at pH values 4.0, 6.8, and 10.0. (**d**) The illustration of the overlapping effect of two spectral components corresponding to deprotonated and protonated forms. ${E}_{R}^{456.6}$ and ${E}_{RH}^{457}$ are enhancements factors for deprotonated and protonated forms at their resonance frequencies, 456.6 MHz and 457 MHz; ${E}_{RH}^{456.6}$ and ${E}_{R}^{457}$ are the corresponding overlap factors (see Eqs (1a) and (1b)).

Monophosphonated perdeuterated trityl radical (dpTAM, Fig. 1a) has been used as a multifunctional paramagnetic probe for concurrent measurement of several tissue parameters (*p*O_2_, pH, and Pi) *in vivo* using L-band EPR spectroscopic approaches^4,21^. dpTAM exhibits a doublet EPR spectrum due to hyperfine splitting on the phosphorus nucleus. dpTAM probe exists in two forms (acidic and basic) at physiological range of pH due to reversible protonation of phosphono group (pK = 6.9). Therefore, four lines are observed in the EPR spectra of dpTAM radical (Fig. 1b) in the pH range from 6 to 8 pH units.

**Figure 2:**
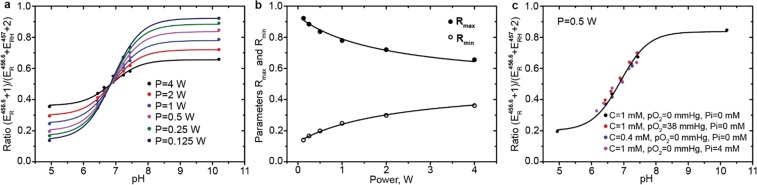
(**a**) The pH dependence of enhancement factor ratio $({E}_{R}^{456.6}+1)/({E}_{R}^{456.6}+{E}_{RH}^{457}+2)$ at different irradiation powers (C = 1 mM, *p*O_2_ = 0 mmHg). The solid lines are the best fits of Eq. (4) to the experimental data yielding the pK value 6.88 ± 0.01. (**b**) Dependence of parameter *R*_*max*_ and *R*_*min*_ (see Eq. (4)) on irradiation power. The solid lines are the best fits of Eq. (5) to the experimental data yielding following values: *s*_1_ = (30.7 ± 2.7) µT^2^; *s*_2_ = (32.7 ± 3.2) µT^2^; *s*_3_ = (4.7 ± 0.5) µT^2^; *s*_4_ = (38 ± 2) µT^2^. (**c**) pH titration curve measured at different dpTAM probe concentrations, *p*O_2_ values, and Pi at irradiation power of 0.5 W.

The high frequency component of dpTAM dynamic nuclear polarization (DNP) spectra at acidic (pH = 4), neutral (pH~7) and basic (pH = 10) pH are shown in Fig. 1c. Similar to EPR spectra (Fig. 1b), DNP spectra of the high frequency component are characterized by a singlet in acidic and basic solutions and a doublet at neutral pH. The ratio of enhancement factors of protonated, ${E}_{RH}^{457}$, and deprotonated, ${E}_{R}^{456.6}$, forms strongly depends on pH value (see Fig. 2a) and can be described by a standard titration curve^39^ (see Eq. (4), Methods Section) with pK value equal to 6.88 in agreement with published data^4,21^. pH titration of the dpTAM probe was performed at different conditions, as shown in Fig. 2a,c. It was found that the titration curve shape significantly depends only on irradiation power level (Fig. 2a,b) while the influence of *p*O_2_, Pi and contrast agent concentration (C) is negligible in the range of pH 6.4–7.4 (Fig. 2c), therefore allowing pH mapping independently of *p*O_2_, Pi and C parameters.

The mapping of *p*O_2_ values and contrast agent concentration using OMRI techniques are well documented for single line EPR trityl probes^24,26,29,35^. However, the multifunctional dpTAM trityl probe high frequency component of the DNP spectrum consists of two partially overlapped spectral components (see Fig. 1d). The semi-empirical mathematical description was developed to correctly describe the dependences of enhancement factor values on oxygen level and contrast agent concentration. Conventional Overhauser enhancement equations were modified by introducing the overlap factors (see Eqs (1a) and (1b), Methods Section) to describe a spectral lines overlapping effect (see Fig. 1d). The contribution of dpTAM probe Heisenberg spin self-exchange^40^ and oxygen-dependent polarization leakage^24^ have been taken into account (see Methods Section for details). The developed approach allows the description of the dependence of enhancement factor values of both deprotonated and protonated forms on irradiation power, C and *p*O_2_ independently. The mathematical description was elaborated using a set of 36 experimental dependences acquired at various pH, *p*O_2_ and spin probe concentrations.

**Figure 3:**
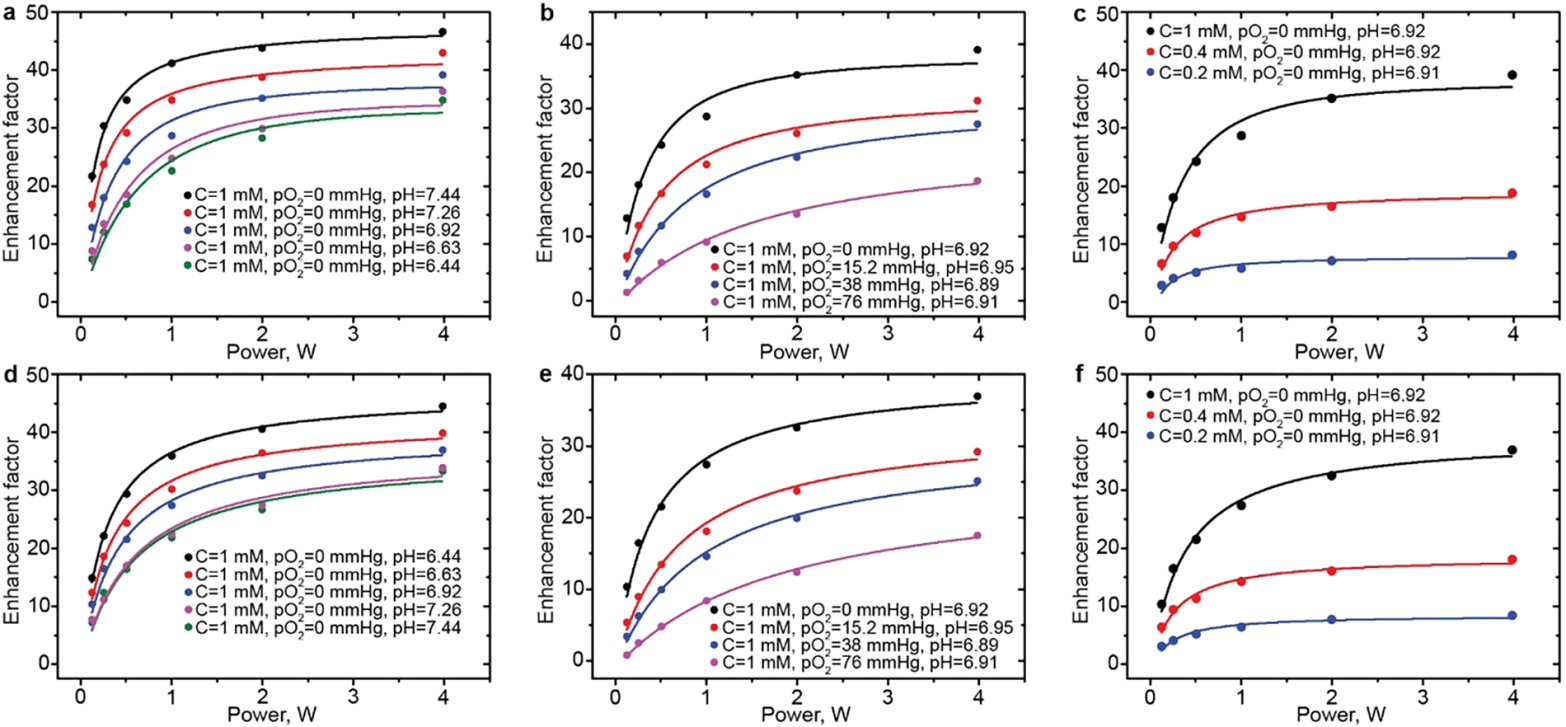
The dependence of enhancement factor on irradiation power measured at different pH (**a,d**), *p*O_2_ (**b,e**) and C (**c,f**) values for protonated (**a–c**) and deprotonated (**d–f**) forms of dpTAM. The solid lines are the best fits of Eqs (1a) and (1b) to the experimental data yielding the values of spin probe concentration, C, and *p*O_2_ listed in the Table SI1 (see Supporting Information). pH values were determined using Eq. (4) for enhancement data obtained at powers 0.125, 0.25, and 0.5 W.

The developed approach for simultaneous pH, *p*O_2_, and C computation was first tested on phantom samples. Figure 3a–f represents the dependencies of enhancement factors, ${E}_{R}^{456.6}$ and ${E}_{RH}^{457}$, on irradiation power at different C, *p*O_2_ and pH values. These dependencies were fitted using Eqs (1a) and (1b) yielding the values of spin probe concentration and *p*O_2_, while pH values were determined independently using Eq. (4). The calculated values of C, *p*O_2_, and pH are listed in Table SI1 (see SI) and are in satisfactory agreement with prepared solution values of C, *p*O_2_, and pH.

**Figure 4:**
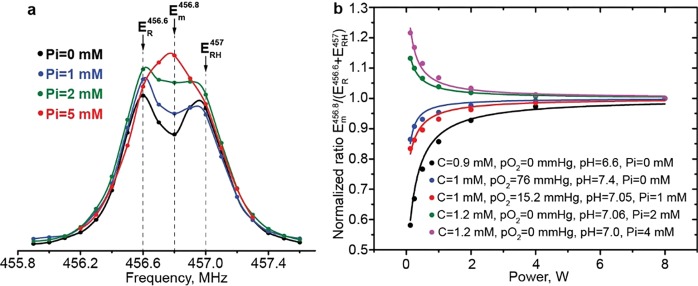
(**a**) The DNP spectrum of the high frequency component of 1 mM dpTAM solution in the presence of phosphate buffer concentrations of 0, 1, 2 and 5 mM at pH 6.9. Spectra were rescaled for a visual illustration of phosphate buffer addition influence. (**b**) Dependence of ratio ${E}_{m}^{456.8}/({E}_{R}^{456.6}+{E}_{RH}^{457})$ on irradiation power, at different concentrations of spin probe, oxygen and phosphate buffers indicated in the Figure. The ratio was normalized on its maximum value at infinite power. The solid lines are the best fits of Eq. (6) to the experimental data yielding the following values of Pi: 0.0 ± 0.1 mM; 0.30 ± 0.03 mM; 1.16 ± 0.04 mM; 2.18 ± 0.01 mM; 4.62 ± 0.04 mM.

The chemical reaction of proton exchange of dpTAM probe with inorganic phosphate results in the coalescence of two resonance lines of protonated and deprotonated forms providing a basis for Pi measurements (see Fig. 4a and ref.^38^). The intensity of the DNP spectrum in the middle point (${E}_{m}^{456.8}$) strongly depends on phosphate buffer concentration (see Fig. 4a). On the other hand, enhancements at protonated (${E}_{RH}^{457}$) and deprotonated (${E}_{R}^{456.6}$) EPR frequencies negligibly depend on Pi in the range 0–4 mM (see Figs S3a,b, SI) allowing for semi-empirical Pi calculation (see Eqs (6) and (7), Methods Section) independently on the values of pH, C, and *p*O_2_ as illustrated in Fig. 4b. The ratio, ${E}_{m}^{456.8}/({E}_{R}^{456.6}+{E}_{RH}^{457})$, measured at different Pi values was normalized on its maximum value at infinite power to reduce the experimental error of enhancement measurement. The normalized ratio dependences on irradiation power were fitted by Eq. (6) (see Methods Section) yielding the calculated Pi concentration values in satisfactory agreement with the prepared ones (see Fig. 4b).

### Multiparametric phantoms imaging

**Figure 5:**
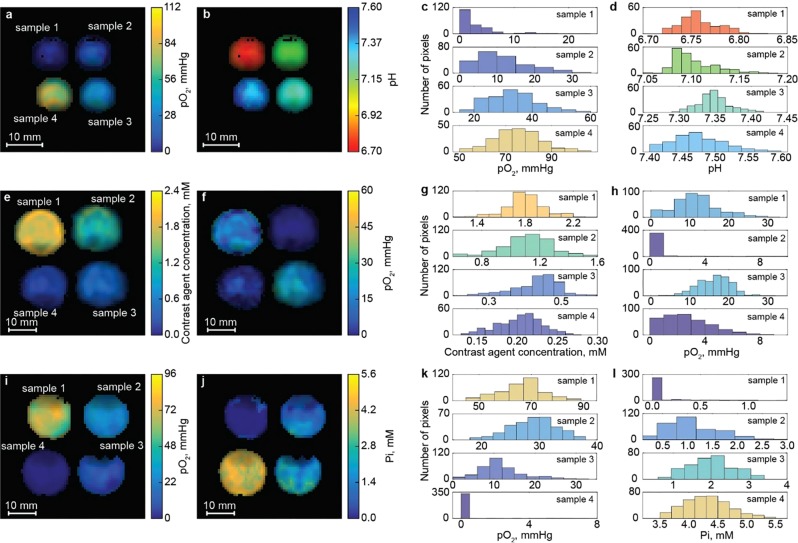
Multifunctional 2D maps and corresponding histograms of the parameter distributions calculated for three phantoms consisting of four glass vials indicated as samples 1–4 and filled with the solutions of different dpTAM probe concentrations, *p*O_2_, pH, and Pi listed in Table 1. Phantom 1. The *p*O_2_ (**a**) and pH (**b**) maps and the histograms of *p*O_2_ (**c**) and pH (**d**) distributions. Phantom 2. The contrast agent concentration (**e**), and *p*O_2_ (**f**) maps and the histograms of these parameters distributions (**g**,**h**, correspondingly). Phantom 3. The *p*O_2_ and Pi maps and the histograms of *p*O_2_ (**k**) and Pi (**l**) distributions. Calculated values of the parameters and their standard deviations for each vial are listed in Table 1. Acquisition parameters are: *T*_*EPR*_, 500 ms; *T*_*R*_, 700 ms; *T*_*E*_, 37 ms; matrix, 64 × 64; field of view, 40 × 40 mm^2^ for the first phantom, and 30 × 30 mm^2^ for the second and third phantoms; slice thickness, 100 mm; 4.2, 5.7 and 5.4 min for phantom samples 1–3, respectively; the simultaneous contrast agent concentration, *p*O_2_ and pH imaging was performed using two powers and two frequencies of EPR irradiation, 456.6 and 457 MHz; the Pi imaging required two additional image acquisitions at EPR frequency of 456.8 MHz measured at two powers; applied powers of EPR irradiation, 0.5 and 4 W, 0.25 and 2 W, 0.5 and 8 W for the first, second and third phantoms, correspondingly; NMR frequency, 686.3 kHz.

To demonstrate the feasibility of simultaneous imaging of multiple parameters, we performed OMRI measurements of several phantom samples to generate 2D maps of C, *p*O_2_, pH, and Pi distributions. In total, mapping of these four parameters requires the acquisition of six OMRI images performed at two EPR powers and three frequencies of EPR irradiation. Note that collection of a conventional MRI (EPR-off) image is required for calculation of all parameters. Figure 5 represents 2D functional maps and corresponding histograms of parameter distributions for three different phantoms of four glass vials filled with dpTAM solutions with different C, *p*O_2_, pH, and Pi values listed in Table 1. As seen in Table 1, the calculated values are in a reasonable agreement with prepared ones.

**Table 1:** Prepared and calculated values of pH, contrast agent concentrations (C), oxygen partial pressures (*p*O_2_) and phosphate buffer concentrations (Pi) and standard deviations (SD) for phantom samples presented in Fig. 5.

phantom	vial	prepared				calculated mean values ± SD			
		pH	C, mM	*p*O_2_, mmHg	Pi, mM	pH	C, mM	*p*O_2_, mmHg	Pi, mM
1	1	6.77	1.2	0	0	6.76 ± 0.02	fixed	3 ± 4	none
	2	7.12	1.2	15.2	0	7.10 ± 0.02	fixed	11 ± 7	none
	3	7.33	1.2	38	0	7.35 ± 0.02	fixed	33 ± 8	none
	4	7.51	1.2	76	0	7.47 ± 0.04	fixed	75 ± 10	none
2	1	7.0	2	15.2	0	6.97 ± 0.02	1.80 ± 0.16	12 ± 6	none
	2	7.0	1	0	0	7.05 ± 0.02	1.1 ± 0.2	0 ± 2	none
	3	7.0	0.4	15.2	0	7.03 ± 0.03	0.43 ± 0.06	16 ± 4	none
	4	7.0	0.2	0	0	7.00 ± 0.03	0.20 ± 0.03	3 ± 2	none
3	1	6.8	1	76	0	6.83 ± 0.03	fixed	66 ± 8	0.1 ± 0.2
	2	6.8	1	38	1	6.79 ± 0.02	fixed	29 ± 4	1.1 ± 0.5
	3	6.8	1	15.2	2	6.74 ± 0.02	fixed	12 ± 5	2.0 ± 0.6
	4	6.8	1	0	4	6.76 ± 0.02	fixed	0.3 ± 1.2	4.3 ± 0.4

The designations: *fixed*, the parameter was fixed as prepared value for calculation procedures; *none*, the parameter was not calculated.

### Multiparametric imaging of normal mouse mammary gland and breast cancer tumor

**Figure 6:**
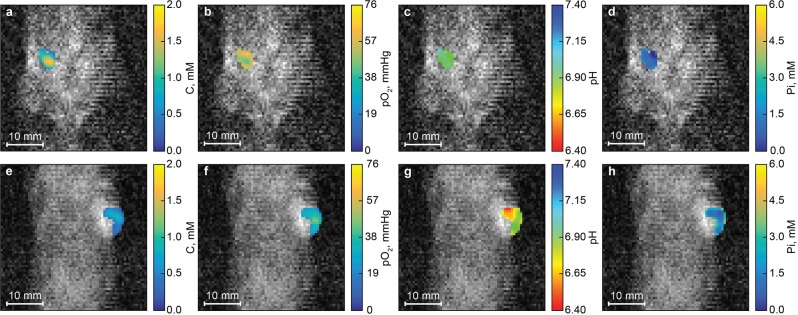
Multiparametric imaging of mouse mammary gland and tumor tissues. (**a**–**d**) The contrast agent, *p*O_2_, pH, and Pi maps of mouse mammary gland. (**e**–**h**) The contrast agent, *p*O_2_, pH, and Pi maps of mouse tumor. Tumor volume of 0.2 cm^3^. Acquisition parameters are: *T*_*EPR*_, 500 ms; *T*_*R*_, 700 ms; *T*_*E*_, 37 ms; matrix, 64 × 64; field of view, 40 × 40 mm^2^; slice thickness, 4 mm; total acquisition time, 4.3 min; imaging performed using powers 0.25 and 8 W and frequencies of EPR irradiation 456.6, 456.8 and 457 MHz; NMR frequency, 686.3 kHz. Mean values and standard deviations of pO_2_, pH and Pi parameters in the normal mammary gland and mammary tumors were found to be equal to 53 ± 6 and 32 ± 7 mmHg, 7.00 ± 0.06 and 6.7 ± 0.1 pH units, 0.9 ± 0.4 and 1.8 ± 0.9 mM, correspondingly. The significant difference (p < 0.05) was found for all (pH, pO_2_, and Pi) parameter distributions measured in normal mammary gland versus mammary tumor tissues.

Concurrent multiparametric imaging of the tissue microenvironment of normal mammary gland performed in anesthetized FVB/N wild type mouse yields C, *p*O_2_, pH, and Pi maps, as shown in Fig. 6a–d. Functional maps were superimposed with MRI image. The mean parameters values and their standard deviations are equal to 1.0 ± 0.4 mM, 53 ± 6 mmHg, 7.00 ± 0.06, 0.9 ± 0.4 mM for C, *p*O_2_, pH and Pi maps, correspondingly.

Multiparametric imaging of the tissue microenvironment of breast cancer tumor performed in MMTV-PyMT transgenic mouse yields functional maps shown in Fig. 6e–h. The mean parameters values for C, *p*O_2_, pH and Pi maps, and their standard deviations are equal to 0.6 ± 0.2 mM, 32 ± 7 mmHg, 6.7 ± 0.1, 1.8 ± 0.9 mM, correspondingly.

**Figure 7:**
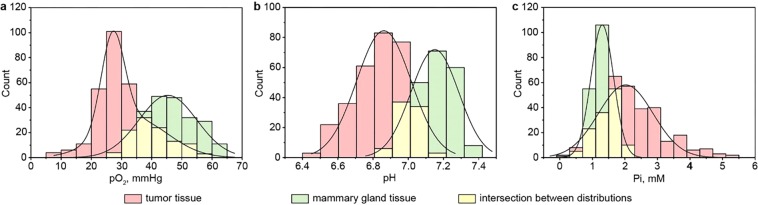
Histograms of *p*O_2_ (**a**), pH (**b**) and Pi (**c**) distribution for mammary gland and tumor tissues. Data were combined from sets of animals. The number of animals was equal to 4 and 7 for mice with breast cancer tumor and healthy mammary gland, correspondingly. The solid lines are the best fits of normal distribution yielding the following mean values and standard deviations 27 ± 4 and 34 ± 11 mmHg of *p*O_2_ (tumor *p*O_2_ distribution represent the mixture of two normal distributions), 6.86 ± 0.15 pH units and 2.0 ± 0.8 mM for [Pi] in tumor tissue; and 45 ± 10 mmHg of *p*O_2_, 7.15 ± 0.14 pH units and 1.3 ± 0.3 mM for [Pi] in mammary gland tissue. The significant difference (p < 0.05) was found for all (pH, pO_2_, and Pi) parameter distributions measured in normal mammary gland versus mammary tumor tissues.

Identical experiments were performed on sets of animals; the total number of mice was 7 and 4 for experiments with healthy mammary gland and tumors, respectively. All obtained multiparametric imaging data and corresponding mean values are presented in SI. Figure 7 shows pO_2_, pH, and Pi histograms for data combined from all images. Histograms were fitted by normal distributions yielding the following mean values of *p*O_2_, pH and Pi and their standard deviations: 27 ± 4 and 34 ± 11 mmHg (tumor *p*O_2_ distribution is a mixture of two normal distributions), 6.86 ± 0.15 and 2.0 ± 0.8 mM in the case of tumor tissue; 45 ± 10 mmHg, 7.15 ± 0.14 and 1.3 ± 0.3 mM in the case of mammary gland tissue.

In a separate experiment, the pharmacokinetics of the dpTAM probe was studied in healthy and tumor-bearing mice. The disappearance of dpTAM signal from a tumor or mammary gland was accompanied by the appearance of dpTAM signal in the mouse bladder (see Fig. S15, SI). The characteristic time (half of signal intensity change) of dpTAM wash out from tissues was calculated to be equal to 26 min for tumor and 43 min for mammary gland. The characteristic time of dpTAM signal accumulation in the bladder was found to be in the range 34–40 min and 58–78 min for tumor and mammary gland, correspondingly.

## Discussion

Noninvasive *in vivo* multifunctional mapping of tumor microenvironment (TME) parameters such as *p*O_2_, pH and Pi may provide a unique insight into understanding mechanisms of tumor pathophysiology and become a useful tool for the development of new therapeutic approaches. However, in the present time, there are no available techniques for concurrent imaging of these parameters. In this work, we present a multiparametric imaging method for concurrent visualization of spin probe concentration, *p*O_2_, extracellular pH, Pi, and anatomical structure using single contrast agent and OMRI technique.

Recently, we designed a monophosphonated trityl spin probe, dpTAM (see Fig. 1a) with EPR spectrum possessing unique multiple functional sensitivity to *p*O_2_, pH, and Pi^4,21,38^. In our previous work, we employed low-field L-band EPR spectroscopy to monitor these parameters in several mouse models of cancer^4^. However, EPR spectroscopic approaches still have the disadvantage of reporting the average values of the TME parameters and does not allow assessing their spatial distribution in tumors characterized by high heterogeneity. To perform multifunctional mapping using a dpTAM probe, we develop an OMRI approach that acquires set of images at different EPR irradiation powers and different EPR frequencies. A minimal number of image acquisitions required for extracting *p*O_2_, pH, Pi, and probe concentration has been justified by testing *in vitro* using phantom samples. Maps of functional parameters have been calculated using the semi-empirical mathematical description of experimental data. The developed OMRI approach allows for multiparametric mapping using six OMRI images obtained at three frequencies and two powers of EPR irradiation. Also a conventional MRI (EPR-off) image has to be collected to retrieve anatomical information and the base level of NMR signal intensity.

Previously, several attempts were performed using OMRI technique to map the acidity using nitroxide^20^ or trityl spin probes^39^. It has been shown that pH mapping can be performed using two OMRI images obtained at irradiation frequencies of protonated and deprotonated forms of pH-sensitive contrast agent. We performed the pH titration of the dpTAM probe and observed dependence of titration curve shape on irradiation power while the influence of *p*O_2_, Pi and contrast agent concentration (C) was insignificant in the range of pH 6.4–7.4 (see Fig. 2a–c). Therefore, in physiological ranges of functional parameters, pH from 6.4 to 7.4, *p*O_2_ from 0 to 80 mmHg, and Pi from 0 to 4 mM, the values of pH can be calculated using only two OMRI images acquired at two different EPR frequencies. The maximum error caused by simplification of titration curve description reaches 0.1 pH units at the edges of considered pH range. Note that the collection of naive MRI images is not required for pH assessment.

Trityl radicals with a single line EPR/DNP spectrum were applied for *p*O_2_ imaging using OMRI technique^24,26,29,35^. The theoretical foundation for these measurements shows that the collection of two OMRI images obtained at two different irradiation powers and one naive MRI (EPR-off) image is sufficient for *p*O_2_ and contrast agent concentration determination. In the case of the dpTAM spin probe, *p*O_2_ measurements can be performed independently using both protonated and deprotonated forms of a probe which potentially increases the accuracy of the *p*O_2_ determination. Thereby, contrast agent concentration and *p*O_2_ mapping can be performed using four OMRI images acquired at two EPR pumping powers at two frequencies of protonated and deprotonated forms of the dpTAM probe. Note that the collection of a naive MRI image is required.

The presented approach for Pi mapping requires OMRI acquisition at frequencies of protonated and deprotonated forms, and at the mid-point frequency most sensitive to the Pi (see Fig. 4a), each at two powers of EPR irradiation. Therefore, multiparametric C, *p*O_2_, pH, and Pi imaging can be performed using six OMRI images and one EPR-off MRI image.

Practically, the application of the developed approach is limited by contrast agent concentration in the range below 2 mM due to the significant contribution of Heisenberg spin self-exchange at high dpTAM probe concentration in the exchange process. High contrast agent concentration results in a high rate of proton exchange between protonated and deprotonated forms of dpTAM^38^, which significantly complicates the discrimination between Pi- and self-exchange contributions.

The developed theoretical semi-empirical foundation allows us to describe the enhancement factors ${E}_{R}^{456.6}$ and ${E}_{RH}^{457}$, their ratios $({E}_{R}^{456.6}+1)/({E}_{R}^{456.6}+{E}_{RH}^{457}+2)$ and ${E}_{m}^{456.8}/({E}_{R}^{456.6}+{E}_{RH}^{457})$, as functions of irradiation power, C, *p*O_2_, pH, and Pi (see Methods Section for details). The developed approach was successfully applied for simultaneous C, *p*O_2_, pH (see Fig. 3a–f), and independent Pi calculations (see Fig. 4b). Multiparametric imaging was performed using three different phantom samples. Figure 5 shows examples of concurrent *p*O_2_ and pH, *p*O_2_ and C, *p*O_2_, and Pi imaging. These experiments show that solving of the multiparameter problem is accompanied by broadening of parameters distribution due to the accumulation of errors. Nevertheless, all calculated parameters are normally distributed and their standard deviation values (see Table 1) are in an acceptable range.

After successful imaging experiments with phantom samples, we performed *in vivo* experiments in mouse normal mammary glands and breast tumors. Figure 6a–d represents maps of functional parameters in mammary gland tissue. Images clearly show uniform distribution of high *p*O_2_, neutral pH, and low Pi values in the region of interest. Concurrent multiparametric mapping of tumor tissue is shown in Fig. 6e–h. Images of tumor tissues are characterized by: (i) approximate uniform hypoxic area compared to *p*O_2_ value of normal tissue; (ii) heterogeneous pH distribution with average pH value significantly lower compared to healthy tissue; (iii) heterogeneous Pi distribution with average Pi value two times higher compared to normal mammary gland. Similar imaging experiments were performed on several normal mammary glands (n = 7) and tumors (n = 4) resulting in the comparable mean values of functional parameters (see Figs S4–S14 and Table SI2). Figure 7 shows *p*O_2_, pH, and Pi distribution for combined data sets of normal and tumor tissues. Data were fitted by normal distribution to elicit the mean values and standard deviations (see Fig. 7). Significant differences were identified for two types of tissue. TME oxygen value distribution covers the large variation in values from hypoxic to normoxic areas and can be described by combination of two normal distributions with mean values considerably lower compared to *p*O_2_ values in mammary gland tissue. pH value distribution for malignant tissue is significantly shifted by approximately 0.3 pH units (p < 0.05, see Fig. 7) toward acidic values compared with normal mammary gland tissue. Interstitial inorganic phosphate concentration in tumor tissue was found to be 2 times higher compared to healthy mammary gland. This observation emphasizes the importance of Pi as a marker of tumor progression^4^. Note that the Pi value distribution width in tumors (SD = 0.8 mM) is significantly broader compared to its mammary gland counterpart (SD = 0.3 mM). In contrast, the widths of pH and *p*O_2_ distributions are comparable for both tumor and mammary gland tissues. The presented results of these pilot animal studies were found to be in agreement with the values previously measured by L-band EPR spectroscopy^4,20^ demonstrating capability of the multifunctional OMRI mapping for *in vivo* studies. The future animal applications of the developed OMRI approach may include investigating relationship between tumor progression and appearance of hypoxic, acidic and high [Pi] areas and correlation between these TME parameters, and may provide new insights into the underlying biochemical mechanisms of tumorigenesis.

The dpTAM trityl probe is a water soluble amphiphilic compound. Its application *in vivo* is limited by local intratissual delivery due to toxicity of “Finland”-type trityls upon systemic delivery^41^. Nevertheless, this probe is characterized by low toxicity by local delivery, in part due to negligible doses used under itratissue delivery (100–1000 times lower compared to i.v. delivery). We have observed slow transfer of dpTAM probe (see Fig. S15) from tissue to the bladder due to wash out of probe by blood flow. The characteristic (half-life) time was found to be in the range of 26–43 minutes, which justifies the insignificant change in dpTAM probe concentration over acquisition time of about 4 minutes. Note that we were not able to estimate bladder pO_2_, pH and Pi content using dpTAM probe due to both the low acidity and high phosphate content of urine.

This work demonstrates a unique capability of the OMRI technique in combination with specially designed trityl spin probe for multiparametric imaging of pH, contrast agent concentration, *p*O_2_ and inorganic phosphate concentration in phantom samples, mouse tumors and normal mammary gland tissues. Based on the enhancement of the proton MRI signal after EPR irradiation of paramagnetic probes, OMRI inherently offers a high spatial resolution, plane selectivity, and rapid image data collection. Previously we demonstrated the capability of OMRI for *in vivo* pH mapping of extracellular pH in tumors in a mouse model of breast cancer using a pH-sensitive imidazoline nitroxide^38^. Note that OMRI applications benefit from using paramagnetic probes with a narrow line width to obtain a high enhancement and low radio frequency power deposition. In the case of the nitroxide probe, the irradiation-induced body temperature increase measured with the microsensor did not exceed 1 °C^38^. Trityl probes possess significantly more narrow linewidth (ΔHpp ≈ 40 mG for dpTAM) compared to the nitroxides (ΔHpp ≈ 1.2 G for imidazoline nitroxide^38^) and at least one order of magnitude longer relaxation times allowing for their easy saturation and application of low specific absorption rates, therefore making them preferable paramagnetic probes for functional *in vivo* pre-clinical OMRI applications^25^. The multifunctional monophosphonated trityl probe, dpTAM, used in this work, is applicable for intratissue delivery only due to its interaction with plasma albumin^41^. Further synthetic work to improve the probe biocompatibility and suitability for systemic delivery is in progress based on two alternative strategies, (i) incorporation of monophosphonate group in core structure of Ox071 trityl probe suitable for systemic delivery^15,25^; and (ii) conjugation of dpTAM probe with a biocompatible carrier, such as dextran^42^. In summary, the multifunctional OMRI technique significantly broadens the area of pre-clinical EPR-based applications allowing for mapping and correlation of physiologically-relevant tissue parameters in various disease models of cancers and beyond. Moreover, as an MRI-based approach, multifunctional OMRI has potential for translation into clinical settings upon progress in instrumentation (e.g., in our previous work^38^ OMRI system based on the whole body 0.38 T clinical scanner was used) and in the development of biocompatible probes.

## Methods

### Contrast agent

Deuterated phosphonated trityl radical (dpTAM) (see Fig. 1a) used as a paramagnetic multifunctional contrast agent was synthesized according to published procedure^21^.

### OMRI scanner, pulse sequence, and experimental parameters

The OMRI experiments were performed on an OMRI desktop imager (Keller JXI-KC02, Japan Redox Ltd.) using a standard fast spin echo sequence for MRI. Imaging experiments were performed in 2D modality. The scanning conditions for the OMRI experiment were as follows: repetition time (*T*_*R*_) × echo time (*T*_*E*_) × EPR irradiation time (*T*_*EPR*_), 700 × 37 × 500 ms; echo factor, 4; frequencies of EPR irradiation, 456.6, 456.8, 457 MHz; slice thickness, 100 mm for phantom imaging, 4 mm for animal imaging; field of view, 30 × 30 mm^2^ or 40 × 40 mm^2^; matrix size, 64 × 64; number of averages was 10 for MRI (EPR-off) images, 2 for OMRI images (for EPR power 8 and 0.25 W) in animal experiments and was varied as 2, 2, 4, 4, 4, 6, 6 for irradiation powers 8, 4, 2, 1, 0.5, 0.25, 0.125 W in experiments with phantom samples. The scanning times of images with two and ten averages were 24 and 114 s, correspondingly. Total time of OMRI + MRI (EPR-off) image set in *in vivo* experiments 2 EPR powers × 3 EPR frequencies × 24 s + MRI (EPR-off) image = 2 × 3 × 24 + 114 = 4.3 min. MRI detection of ^1^H water signal was performed on field 0.016 T (0.686 MHz).

### Specific absorption rate estimation for OMRI experiment

Specific absorption rate (SAR) was estimated by measurement of the Q-factor of empty and mouse-loaded resonator^43^. It was found that 23.5% of applied power was absorbed by the mouse body (weight 36 g). Taking into account the duty cycle of the EPR irradiation (500 ms in 700 ms or 71.4%) with two different powers (0.25 and 8 W) the estimated average SAR was 19.2 W·kg^−1^. A series of six OMRI images were performed over 2.4 minutes by alternating sequences with the high (8 W) and the low (0.25–0.5 W) irradiation powers, each 24 s, to avoid a body heating of the mice. The animal temperature was measured using Nomad Fiber Optic Thermometer (Neoptix, Canada) equipped with Fiber Optic Temperature Sensor with a probe diameter of 0.5 mm, placed on the shaved skin chest area. The measured temperature did not exceed 37 °C during the entire OMRI experiment. All animals were alive until the end of the experiment and placed back to the cage after the end of anesthesia.

### Theory and calibration procedures

The enhancement factors for deprotonated and protonated forms are described by equations (see SI for the details):

1a $${E}_{R}^{456.6}(P)=|1-{E}_{R}^{inf}\cdot f\cdot {S}_{R}(P)|+{E}_{RH}^{456.6}(P),$$

1b $${E}_{RH}^{457}(P)=|1-{E}_{RH}^{inf}\cdot f\cdot {S}_{RH}(P)|+{E}_{R}^{457}(P),$$

where indexes *R* and *RH* correspond to deprotonated and protonated forms of dpTAM; ${E}_{R}^{456.6}$ and ${E}_{RH}^{457}$ are the enhancement factors of deprotonated and protonated forms measured at their resonance frequencies 456.6 and 457 MHz of the high frequency component of the DNP spectrum (see Fig. 1c); ${E}_{R}^{inf}$ and ${E}_{RH}^{inf}$ are the enhancement factors of deprotonated and protonated forms at infinite power and contrast agent concentration (${E}_{R,RH}^{inf}=({\gamma }_{S}/{\gamma }_{I})\cdot {\varepsilon }_{R,RH}$, where *ε*_*R*_ and *ε*_*RH*_ are the coupling factors, *γ*_*S*_ and *γ*_*I*_ are the values of the gyromagnetic ratio of electron and proton); *f* is the leakage factor; *S*_*R*,*RH*_ is the saturation factor, ${E}_{RH}^{456.6}$ and ${E}_{R}^{457}$ are the overlap factors – the enhancement factors of protonated and deprotonated forms determined at resonance frequencies 456.6 and 457 MHz (see Fig. 1d); *P* is a power of EPR irradiation. Theoretical details of enhancement factors description are provided in SI.

The leakage factor can be described as following^24^:

2 $$f={f}_{0}\cdot {f}_{l}=\frac{r\cdot C\cdot {T}_{10}}{1+r\cdot C\cdot {T}_{10}}\cdot \frac{1}{1+{T}_{1}\cdot {w}_{l}^{0}\cdot p{O}_{2}},$$

where *T*_10_ and *T*_1_ are the relaxation times of water protons in the absence and the presence of spin probe, *r* is a relaxivity constant, ${w}_{l}^{0}$ is oxygen-induced leakage rate constant, *f*_0_ is a traditional interpretation of leakage factor, *f*_*l*_ a part of the leakage factor which depends on the concentration of the paramagnetic impurity (e.g., oxygen).

The second parameter in enhancement factors descriptions (1a) and (1b) is the saturation factor^40^:

3 $$\begin{array}{rcl}{S}_{R}(P) & = & \frac{1}{2}\cdot \frac{\alpha \cdot P}{{g}_{R}^{456.6}+\alpha \cdot P\cdot {f}_{exc}^{R}},\,{S}_{RH}(P)=\frac{1}{2}\cdot \frac{\alpha \cdot P}{{g}_{RH}^{457}+\alpha \cdot P\cdot {f}_{exc}^{RH}},\\ {f}_{exc}^{R,RH} & = & \frac{1+C\cdot {w}_{exc}\cdot {({T}_{1e})}_{R,RH}}{1+2\cdot C\cdot {w}_{exc}\cdot {({T}_{1e})}_{R,RH}},\,{g}_{R,\,RH}^{456.6,\,457}={(\frac{1}{{\gamma }_{S}^{2}{T}_{1e}{T}_{2e}})}_{R,RH},\end{array}$$

where a numerical factor $\frac{1}{2}$ represents a saturation of only high frequency component of the DNP spectrum of the dpTAM probe, *α* is a resonator efficiency factor measured for each sample in a separate experiment, *T*_1*e*_ and *T*_2*e*_ are the electron spin relaxation times of the contrast agent, ${f}_{exc}^{R,RH}$ are parameters represented the influence of Heisenberg spin exchange on the shape of the DNP spectrum (self-exchange of dpTAM probe results in ‘preservation’ of spin probe polarization), *w*_*exc*_ is the rate constant of spin exchange. A detailed description of parameters ${g}_{R}^{456.6}$, ${g}_{RH}^{457}$, *w*_*exc*_ and overlap factors ${E}_{RH}^{456.6}\,$and ${E}_{R}^{457}$ are provided in SI.

The ratio of enhancement factors of protonated and deprotonated forms strongly depends on pH value and can be described by the standard titration curve^38,39^:

4 $$\frac{{E}_{R}^{456.6}(P)+1}{{E}_{R}^{456.6}(P)+{E}_{RH}^{457}(P)+2}=\frac{{R}_{max}(P)+{R}_{min}(P)\cdot {10}^{pK-pH}}{1+{10}^{pK-pH}},\,$$

where *R*_*max*_ and *R*_*min*_ are the maximum and minimum values of the titration curve function. The dependences of *R*_*max*_ and *R*_*min*_ parameters on irradiation power are described by the following equations (see SI):

5 $${R}_{max}(P)=\frac{{s}_{1}+\alpha \cdot P}{{s}_{2}+2\alpha \cdot P},\,{R}_{min}(P)=\frac{{s}_{3}+\alpha \cdot P}{{s}_{4}+2\alpha \cdot P},$$

where *s*_1_, *s*_2_, *s*_3_, *s*_4_ are experimentally calibrated parameters.

The ratio of enhancement factors ${R}_{m}(P)={E}_{m}^{456.8}/({E}_{R}^{456.6}+{E}_{RH}^{457})$, where ${E}_{m}^{456.8}$ is an enhancement factor measured at frequency 456.8 MHz (see Fig. 4a), was used for Pi calculations. The following semi-empirical equation was obtained to describe *R*_*m*_(*P*) as function:

6 $${R}_{m}(P)/{R}_{m}^{max}=\frac{{g}_{+}+\alpha \cdot P}{{g}_{m}+\alpha \cdot P}\,,$$

where are ${R}_{m}^{max}={R}_{m}(P\to \infty )$ is an *R*_*m*_ value at infinite power, parameters *g*_+_ and *g*_*m*_ are described as follows:

7 $$\begin{array}{rcl}{g}_{+} & = & {({b}_{0}+C\cdot ({b}_{1}+{b}_{2}\cdot {10}^{pK-pH})/(1+{10}^{pK-pH})+{b}_{3}\cdot p{O}_{2})}^{2},\\ {g}_{m} & = & {b}_{4}\cdot {g}_{+}-Pi\cdot \frac{|{b}_{5}-({b}_{6}-\,{b}_{7}\cdot Pi)\cdot p{O}_{2}+({b}_{8}-{b}_{9}\cdot Pi)\cdot C|}{1+{10}^{{b}_{10}-pH}},\end{array}$$

where *b*_0_, *b*_1_, *b*_2_, *b*_3_,*b*_4_, *b*_5_, *b*_6_, *b*_7_, *b*_8_, *b*_9_ and *b*_10_ are experimentally calibrated parameters.

Descriptions of calibration procedures are provided in SI. All presented data processing procedures were made using specially developed MATLAB code.

### Calculation procedure in an imaging modality

Enhancement factors (1a) and (1b) depends on C, *p*O_2_, and pH. Parameters C and *p*O_2_ were calculated by a finding a numerical solution of the system of four Eqs (1a) and (1b) using two different irradiation powers *P*_1_ and *P*_2_. (Note that acquisition of third MRI image with no EPR irradiation power is required to calculate the enhancement values). The pH values were calculated solving system of two Eq. (4) at powers *P*_1_ and *P*_2_, obtained pH values were used as parameters for calculation of contrast agent concentrations and oxygen partial pressures.

Phosphate buffer concentrations, Pi, were calculated using a system of two ratios of enhancement factors ${E}_{m}^{456.8}/({E}_{R}^{456.6}+{E}_{RH}^{457})$ obtained at two powers *P*_1_ and *P*_2_ and the highest power (*P*_2_) equal to 8 W was used for estimation of maximum values ${R}_{m}^{max}$:

8 $$\begin{array}{ccccc}\frac{{E}_{m}^{456.8}({P}_{1})}{{E}_{R}^{456.6}({P}_{1})+{E}_{RH}^{457}({P}_{1})}\cdot \frac{{E}_{R}^{456.6}({P}_{2}=8)+{E}_{RH}^{457}({P}_{2}=8)}{{E}_{m}^{456.8}({P}_{2}=8)} & = & \frac{{R}_{m}({P}_{1})}{{R}_{m}^{max}},\frac{{R}_{m}({P}_{2}=8)}{{R}_{m}^{max}} & = & 1,\end{array}$$

where function ${R}_{m}(P)/{R}_{m}^{max}$ is described by Eq. (6), values of parameters C, *p*O_2_, and pH were obtained independently by procedure described above and used for Pi calculation as parameters. Systems of equations were solved numerically in each pixel. Only pixels with enhancement factor higher than one were taken into account for calculation of parameter maps. Calculations were made using specially developed MATLAB code.

### Phantom samples preparation and imaging

The contrast agent was dissolved in 150 mM NaCl solution for samples preparation. Sample solutions were bubbled for 15 minutes with a gas mixture of nitrogen and oxygen in various proportions to provide desired oxygen concentration. pH values were regulated by the addition of HCl or NaOH. All measurements were performed at 33 °C. All calibration samples were constructed using one 8 mL glass vial filled with 4–5 mL of contrast agent solution and one 8 mL glass vial filled with 150 mM NaCl solution. OMRI data were acquired at irradiation powers 0, 0.125, 0.25, 0.5, 1, 2, 4 and 8 W for calibration experiments and mean data values of OMRI images from regions of interest for were used for further data processing. Imaging phantom samples were constructed using four 2 ml glass vials filled with solutions with various spin probe concentration and *p*O_2_, pH, Pi values.

### Animals imaging *in vivo*

All animal work was performed in accordance with the WVU IACUC approved protocol. All experimental procedures were approved by West Virginia University Institutional Animal Care and Use Committee. MMTV-PyMT (polyoma virus middle T antigen) transgenic mice (PyMT+/−) on the FVB/N background (Jackson Laboratories, 002374, FVB/N-Tg (MMTV-PyVT)634Mul/J) which spontaneously-develop breast cancer (4 animals) were used for *in vivo* experiments. For comparison of tissue microenvironments of normal mammary glands and tumors, age-matched littermate females (7 animals) absent of the PyMT oncogene (PyMT−/−) were used. A stock solution of 5–15 mM contrast agent (pH = 7.1) in 150 mM sodium chloride solution was prepared for injection. Injection solution was bubbled slowly during 30 minutes with a gas mixture of nitrogen and oxygen in proportion 93%/7% to provide faster equilibration of tissue microenvironment after injection. Animals were placed into the OMRI resonator and anesthetized by inhalation of the air-isoflurane mixture using DRE VP3 (DRE veterinary, USA) anesthetic machine. A tube with a gas mixture was connected to the rear side of resonator to provide continuous gas supply during the experiment. After the onset of anesthesia, a 50–100 µl of stock solution of contrast agent was injected locally into mouse mammary gland or breast tumor. Fifteen minutes after injection, one MRI and six OMRI images were acquired at irradiation powers 0.25 (or 0.5) and 8 W and frequencies 456.6, 457, 456.8 MHz. Slice thickness of 4 mm was selected so that only signals from which an area that contrast agent contributed to the enhancement of MRI signal was included. Tumor volume was calculated using 2D MRI image measurements of length (L) and width (W) of tumor and equation, V = [L × W^2^] × 0.5, where V is tumor volume^44^.

### Statistical analysis

Statistical analysis was performed using the OriginPro software package. To compare two distributions, the ANOVA test (for normally distributed populations) and non-parametric Mann–Whitney *U* test (for non-normally distributed populations) were used.

## Supplementary information

Supplement Information

## Data Availability

The datasets generated and/or analyzed during the current study are available from the corresponding author on reasonable request.
