# Supplementary material for: Development of multifunctional Overhauser-enhanced magnetic resonance imaging for concurrent in vivo mapping of tumor interstitial oxygenation, acidosis and inorganic phosphate concentration
Source: Sci Rep. 2019 Aug 20;9:12093. doi: 10.1038/s41598-019-48524-3 (PMC6702349; doi:10.1038/s41598-019-48524-3)
Supplement: Supplementary file 1 — Supplement Information [file 41598_2019_48524_MOESM1_ESM.pdf]

Supplement Information

Development of multifunctional Overhauser-enhanced magnetic resonance imaging for concurrent in vivo mapping of tumor interstitial oxygenation, acidosis and inorganic phosphate concentration.

Artem A. Gorodetskii[a,b,c,d], Timothy D. Eubank[a,e,g], Benoit Driesschaert[a,f,g], Martin Poncelet[a,f], Emily Ellis[e], Valery V. Khramtsov[a,b,g]*, Andrey A. Bobko[a,b,g]*

[a]*In Vivo Multifunctional Magnetic Resonance center, Robert C. Byrd Health Sciences Center, West Virginia University, Morgantown, WV 26506, USA*

[b]*Department of Biochemistry, West Virginia University School of Medicine, Morgantown, WV 26506, USA*

[c]*N.N. Voroztsov Novosibirsk Institute of Organic Chemistry SB RAS, Novosibirsk, 630090, Russia*

[d]*Novosibirsk State University, Novosibirsk, 630090, Russia*

[e]*Department of Microbiology, Immunology & Cell Biology, West Virginia University School of Medicine, Morgantown, WV 26506, USA*

[f]*Department of Pharmaceutical Sciences, West Virginia University, School of Pharmacy, Morgantown, WV 26506, USA*

[g]*West Virginia University Cancer Institute, Morgantown, WV 26506, USA*

*Corresponding authors

*E-mail addresses: andrey.bobko@hsc.wvu.edu; valery.khramtsov@hsc.wvu.edu

Theory

Theoretical details of enhancement factors description. The enhancement factors for dpTAM probe, similar to single line trityl probes, might be written as following[1]:

$$E = |I_z/I_0| = |1 - E_{inf} \cdot f \cdot S|, \tag{1}$$

where E is an enhancement factor of NMR signal under EPR irradiation, I_z and I_0 are nuclear spin polarization in the presence and absence of EPR irradiation, E_{inf} is the enhancement factor at infinite power and contrast agent concentration ($E_{inf} = (\gamma_S/\gamma_I) \cdot \varepsilon$, where ε is a coupling factor, γ_S and γ_I are the values of gyromagnetic ratio of electron and proton), f is a leakage factor, S is a saturation factor.

The distance between two DNP spectral lines corresponding to protonated and deprotonated forms of radical dpTAM is less than their linewidths which results in the overlap of DNP spectra of acidic and basic forms (see Fig. 1d, *Main Text*). The semi-empirical mathematical model was developed to describe enhancement factors dependences on applied EPR power and microenvironment parameters. OMRI enhancement factors for acidic and basic forms of the dpTAM can be described by the following equations (see Fig. 1d, *Main Text*):

$$E_R^{456.6} = \left|1 - E_R^{inf} \cdot f \cdot S_R\right| + E_{RH}^{456.6}, \quad (2a)$$

$$E_{RH}^{457} = \left|1 - E_{RH}^{inf} \cdot f \cdot S_{RH}\right| + E_R^{457}, \quad (2b)$$

where are indexes R and RH correspond to deprotonated and protonated forms of radical dpTAM, $E_R^{456.6}$ and E_{RH}^{457} are the enhancement factors of deprotonated and protonated forms measured at their resonance frequencies 456.6 and 457 MHz (high frequency components of the DNP spectrum (see Fig. 1c and 1d, *Main Text*)), E_R^{inf} and E_{RH}^{inf} are the enhancement factors at infinite power and contrast agent concentration ($E_{R,RH}^{inf} = (\gamma_S/\gamma_I) \cdot \varepsilon_{R,RH}$, where ε_R and ε_{RH} are the coupling factors), f is the leakage factor, $S_{R,RH}$ is the saturation factor, $E_{RH}^{456.6}$and E_R^{457} are the overlap factors (see Fig. 1d, *Main Text*).

The presence of paramagnetic impurities such as oxygen stimulates the leakage of spin polarization. Leakage factor can be described as following[1,2]:

$$f = f_0 \cdot f_l = \frac{r \cdot C \cdot T_{10}}{1 + r \cdot C \cdot T_{10}} \cdot \frac{1}{1 + T_1 \cdot w_l^0 \cdot pO_2}, \quad (3)$$

where T_1 and T_{10} are the relaxation times of water protons in the absence and the presence of the spin probe, r is the relaxivity constant, w_l^0 is the leakage rate constant, f_0 is the traditional interpretation of leakage factor, f_l is the part of leakage factor which depends on the concentration of the paramagnetic impurity (e.g., oxygen), C and pO_2 are the spin probe concentration and oxygen partial pressure (see ref.[2] for details).

The second important parameter in the description of the enhancement of signal is the saturation factor S[3]:

$$S_R = \frac{1}{2} \cdot \frac{\alpha \cdot P}{g_R^{456.6} + \alpha \cdot P \cdot f_{exc}^{R,RH}}, S_{RH} = \frac{1}{2} \cdot \frac{\alpha \cdot P}{g_{RH}^{457} + \alpha \cdot P \cdot f_{exc}^{R,RH}},$$
$$f_{exc}^{R,RH} = \frac{1 + C \cdot (w_{exc} \cdot T_{1e})_{R,RH}}{1 + 2 \cdot C \cdot (w_{exc} \cdot T_{1e})_{R,RH}}, g_{R,RH} = \left(\frac{1}{\gamma_S^2 T_{1e} T_{2e}}\right)_{R,RH}, \quad (4)$$

where a numerical factor $\frac{1}{2}$ represents a saturation of only high frequency component of the DNP spectrum of the dpTAM probe, α is the resonator efficiency factor, P is a power of EPR irradiation, T_{1e} and T_{2e} are the electron spin relaxation times of the contrast agent, w_{exc} is the rate constant of Heisenberg spin exchange (self-exchange of dpTAM probe results in 'preservation' of spin probe polarization).

In the case when only one form of radical (protonated or deprotonated) is observed, the influence of contrast agent and oxygen on relaxation times can be expressed as[4]:

$$1/(\gamma_S^2 T_{1e} T_{2e}) = (a_0 + a_1 \cdot C + a_2 \cdot pO_2)^2\,, \quad (5)$$

where a_0, a_1, a_2 are experimentally calibrated parameters.

In the case of intermediate pH (6<pH<8) when both forms (protonated and deprotonated) present in the solution, their concentrations can be found using the following expressions:

$$C_R = C/(1+10^{pK-pH}),$$

$$C_{RH} = C\cdot 10^{pK-pH}/(1+10^{pK-pH}), \tag{6}$$

where C_R and C_{RH} are concentrations of deprotonated and protonated forms of dpTAM radical, pK is the ionization constant of dpTAM radical.

Parameters $g_R^{456.6}$ and g_{RH}^{457} (see eqns. (4)) can be rewritten using equations (5) and (6):

$$g_R^{456.6} = (a_{10} + a_{11}\cdot C/(1+10^{pK-pH}) + a_{12}\cdot pO_2)^2, \tag{7a}$$

$$g_{RH}^{457} = (a_{20} + a_{21}\cdot C\cdot 10^{pK-pH}/(1+10^{pK-pH}) + a_{22}\cdot pO_2)^2. \tag{7b}$$

The rate of Heisenberg spin exchange (dpTAM probe self-exchange) is depending on the pH value of the solution and can be expressed as follows:

$$w_{exc}(pH) = \frac{k_1\cdot 10^{-2pK} + k_2\cdot 10^{-2pH} + k_{12}\cdot 10^{-pH-pK}}{(10^{-pK}+10^{-pH})^2}, \tag{8}$$

k_1, k_2 and k_{12} the spin exchange rate constants for deprotonated and protonated forms of radical and for exchange them to each other.

The overlap factors $E_{RH}^{456.6}$ and E_R^{457} can be expressed as:

$$E_{RH}^{456.6} = (a_5\cdot C/(1+10^{pK-pH}))\cdot f_l\cdot\frac{\alpha\cdot P}{g_{RH}^{456.6}+\alpha\cdot P},$$

$$E_R^{457} = (a_6\cdot C\cdot 10^{pK-pH}/(1+10^{pK-pH}))\cdot f_l\cdot\frac{\alpha\cdot P}{g_R^{457}+\alpha\cdot P}, \tag{9}$$

where parameters $g_{RH}^{456.6}$ and g_R^{457} are described by equations (7b) and (7a) with coefficients a_{30}, a_{31}, a_{32} and a_{40}, a_{41}, a_{42}, respectively.

Several empirical dependences were introduced into the proposed model for improving fitting quality. The following equation was used to describe leakage of spin polarization due to interaction with oxygen in equations (2, 9): $f_l = (1+T_{10}\cdot w_l^0\cdot pO_2)/(1+2\cdot T_{10}\cdot w_l^0\cdot pO_2)$. The following additional members were added into equations (7a) and (7b): $(a_{13}C + a_{14}C^2)10^{pK-pH}/(1+10^{pK-pH})$ and $(a_{23}C + a_{24}C^2)/(1+10^{pK-pH})$, respectively. Parameters a_{ij} are experimentally calibrated. The values of spin exchange constants k_1, k_2 and k_{12} were taken from literature data[5].

Also, the maximum enhancement values for deprotonated and protonated forms calculated at infinite power in basic or acid conditions might be written as follows[4]:

$$E_{R,RH}^{max} = E_{R,RH}(P\to\infty) = \left|1 - \frac{1}{2}\cdot E_{RH}^{inf}\cdot f\cdot\frac{1+2\cdot C\cdot k_{1,2}\cdot(T_{1e})_{R,RH}}{1+C\cdot k_{1,2}\cdot(T_{1e})_{R,RH}}\right|. \tag{10}$$

Theoretical details for description of the pH titration curve. The ratio of enhancement factors of deprotonated and protonated forms strongly depends on pH value[5,6]:

$$\frac{E_R^{456.6}+1}{E_R^{456.6}+E_{RH}^{457}+2}=\frac{R_{max}+R_{min}\cdot 10^{pK-pH}}{1+10^{pK-pH}}. \tag{11}$$

The enhancement factors $E_R^{456.6}$ and E_{RH}^{457} depend on the power of irradiation, pH, oxygen, and contrast agent concentrations. Thereby, parameters R_{max} and R_{min} are the functions of P, C, pO_2. To obtain equations for R_{max} and R_{min} equations (2) were substituted to (11). Significant simplifications were made for qualitative description of parameters R_{max} and R_{min}: (i) the overlap factors in equations (2) were neglected; (ii) it was supposed that $E_{inf}^R = E_{inf}^{RH}$, $a_{10} = a_{20}$, $a_{11} = a_{21}$ and $a_{12} = a_{22}$; (iii) members of equation which are proportional to C^2 and $C \cdot pO_2$ were neglected. Thereby taking into account these simplifications the following equations for parameters R_{max} and R_{min} can be obtained:

$$R_{max}=\frac{(a_0+a_2\cdot pO_2)^2+\alpha P}{2(a_0+a_2\cdot pO_2)^2+2a_0a_1C+2\alpha P},$$
$$R_{min}=\frac{(a_0+a_2\cdot pO_2)^2+2a_0a_1C+\alpha P}{2(a_0+a_2\cdot pO_2)^2+2a_0a_1C+2\alpha P}, \tag{12}$$

It was found that the oxygen, contrast agent and inorganic phosphate concentration does not significantly affect the titration curve of the dpTAM probe (see Figure 2c, *Main Text*) in the range of physiological important pH values between 6.4 and 7.4. Therefore, the influence of the pO_2, C and Pi parameters on the titration curve were neglected:

$$R_{max}(P)=\frac{s_1+\alpha P}{s_2+2\alpha P},$$
$$R_{min}(P)=\frac{s_3+\alpha P}{s_4+2\alpha P}, \tag{13}$$

where s_1, s_2, s_3, s_4 are experimentally calibrated parameters.

Theoretical details of P_i computation. It has been observed that the enhancement ratio $R_m(P) = E_m^{456.8}/(E_R^{456.6} + E_{RH}^{457})$ normalized on its value at maximum power ($R_m^{max} = R_m(P \to \infty)$) strongly depends on irradiation EPR power value. Ratio $R_m(P)/R_m^{max}$ can be satisfactorily fitted with rational function (see Fig. 4b, *Main Text*):

$$R_m(P)/R_m^{max}=\frac{g_+ + \alpha\cdot P}{g_m+\alpha\cdot P}, \tag{14}$$

where g_+ and g_m are fitting parameters.

The following simplifications in the enhancement factor equations were done for the semi-empirical mathematical description of fitting parameters g_+ and g_m: (i) overlap factors (eqn. 2) were neglected; (ii) the $E_{inf}^R \approx E_{inf}^{RH}$ was assumed; (iii) the enhancement factor for the middle point was expressed similar to enhancement factors of protonated and deprotonated forms:

$$E_m^{456.8}=\left|1-E_{inf}^m\cdot f\cdot S_m\right|, \tag{15}$$

$$S_m = \frac{\alpha \cdot P}{g_m + \alpha \cdot P}.$$

Then the ratio $E_m^{456.8}/(E_R^{456.6} + E_{RH}^{457})$ can be expressed as:

$$\frac{E_m^{456.8}}{E_R^{456.6} + E_{RH}^{457}} = \frac{E_{inf}^m}{E_{inf}^R} \cdot \frac{g_R g_{RH} + \alpha P(g_R + g_{RH}) + \alpha^2 P^2}{g_m(g_R + g_{RH}) + \alpha P(g_R + g_{RH} + 2g_m) + 2\alpha^2 P^2}. \quad (16)$$

Similarly to the previous semi-empirical description of pH titration curve, it can be assumed that $a_{10} = a_{20}$, $a_{12} = a_{22}$, $a_{11} \sim a_{21}$ and contribution of members proportional to C^2 is insignificant. In these conditions, the following equation takes place:

$$g_+ = (g_R + g_{RH})/2 \approx (b_0 + C \cdot (b_1 + b_2 \cdot 10^{pK-pH})/(1 + 10^{pK-pH}) + b_3 \cdot pO_2)^2. \quad (17)$$

Also, the following estimation was proposed: $g_R \cdot g_{RH} \sim g_+^2$. Then the equation (16) can be simplified to equation (14) that is more convenient for experimental data analysis.

Figure 4c (*Main Text*) shows the dependence of the ratio $R_m(P)/R_m^{max}$ on irradiation power at the different phosphate buffer concentrations. This ratio is an increasing function at low phosphate concentrations and a decreasing function at high phosphate concentrations. The behavior of the function $R_m(P)/R_m^{max}$ is determined by the sign of its derivative:

$$\frac{\partial\, R_m(P)/R_m^{max}}{\partial P} = \frac{\alpha \cdot \triangle g}{(g_m + \alpha \cdot P)^2}, \quad (18)$$

$$\triangle g = g_m - g_+.$$

Thereby the following two statements can be composed:

$$\triangle g > 0, \quad low\ rate\ of\ exchange,$$
$$\triangle g < 0, \quad high\ rate\ of\ exchange. \quad (19)$$

To satisfy requirements in eqn. (19) ($\triangle g > 0\ at\ Pi \leq 1mM$) we constrained a value to $g_m(Pi = 0) = b_3 \cdot g_+$, where $b_3 > 1$. The high phosphate level results in a negative sign of the first derivative in eqn. (18), therefore:

$$g_m = b_4 \cdot g_+ - g_{Pi}(C, pO_2, pH, Pi) \quad (20)$$

and $g_{Pi} = Pi \cdot g_{Pi}^0$ ($g_{Pi} = 0\ at\ Pi = 0\ mM$). The following equation for parameter g_{Pi} was empirically obtained by analysis of experimental data (see Fig S2a-c):

$$g_{Pi}(C, pO_2, pH, Pi) = P_i \cdot g_{Pi}^0(C, pO_2, pH, Pi), \quad (21a)$$

$$g_{Pi}^0(C, pO_2, pH, Pi) = \frac{h(C, pO_2, Pi)}{1 + 10^{b_{10} - pH}}, \quad (21b)$$

$$h(C, pO_2, Pi) = |b_5 - (b_6 - b_7 \cdot Pi) \cdot pO_2 + (b_8 - b_9 \cdot Pi) \cdot C|, \quad (21c)$$

where b_i are experimentally calibrated parameters.

Calibration and calculation procedures

Proton relaxation time and relaxivity constant. Parameters T_{10} and r were determined by the saturation recovery experiment with samples with different contrast agent concentrations (0, 0.5, 1, 2 mM) in anaerobic conditions at pH 10.0. It has found that values of T_{10} = 3.9±0.1 s and r = 0.15±0.01 $(\mathrm{mM\cdot s})^{-1}$ in a good agreement with published data[7].

Coupling factors. The coupling factors ε_R and ε_{RH} were obtained using the following procedure. The dependence of enhancement factors on microwave power was obtained at the different concentrations of the spin probe in anaerobic conditions (see Figures S1a and S1b) for protonated and deprotonated forms of radical at pH equal 5.0 and 10.0, respectively. The dependences were fitted using the following equation to extract the maximum enhancement values E_{max}[3]:

$$E = -1 + A \cdot P/(1 + B \cdot P),$$
$$E_{max} = -1 + A/B. \qquad (22)$$

The dependences of $E_{R,RH}^{max}$ on contrast agent concentration (see Figure S1c) was approximated by equation (10) yielding the values of the coupling factors ε_R and ε_{RH}, equal to 0.40±0.02 and 0.33±0.01 and enhancements at infinite power and contrast agent concentration, E_R^{inf} and E_{RH}^{inf} , equal to 270±4 and 221±5. These findings agreed to the corresponding parameters measured for "Finland" trityl probe[7].

Resonator efficiency factor. The resonator efficiency factor α was measured for each sample using a time domain sensor (SPEAG) as a proportional coefficient between the square of the magnetic field B_1 and the EPR irradiation power. The resonator efficiency factor α was measured to0 be in the range 7.7-14.2 $\mu T^2/W$ and 6.3-7.3 $\mu T^2/W$ for *in vitro* and *in vivo* experiments, correspondently.

Calibration parameters for enhancement factors. Parameters a_{ij} and w_l^0 were found using the global fitting procedure. The set of experimental data of measured signal enhancements at different irradiation powers (0.125, 0.25, 0.5, 1, 2, 4 W), pH (in the range 6.4-7.4), contract agent (0.2, 0.4, 1, 2 mM) and oxygen concentrations (0, 7.6, 15.2, 38, 76 mmHg) was analyzed using equations (2a) and (2b). To perform global fitting procedure and calculate a_{ij} and w_l^0 values, we have collected the data set of 36 enhancement dependences on EPR power for protonated and deprotonated dpTAM forms at different pH, pO_2 and C. In order to minimize number of unknown constants the following parameters describing influence of oxygen on relaxation rates considered equal to each other: a_{12} and a_{32}; a_{22} and a_{42}. As a result, using the global fitting procedure the following values of parameters were obtained: a_{10}, 1.0 μT; a_{11}, -0.55 μT/mM; a_{12}, 29.5×10^{-3} μT/mmHg; a_{13}, 2.1 μT/mM; a_{14}, 0.37 μT/mM^2; a_{20}, 1.2 μT; a_{21}, -0.55 μT/mM; a_{22}, 29.5×10^{-3} μT/mmHg; a_{23}, 0.85 μT/mM; a_{24}, 0.5 μT/mM^2; a_{30}, -0.85 μT; a_{31}, 9 μT/mM; a_{40}, 0.25 μT; a_{41}, 3.0 μT/mM; a_5, 18.5 mM^{-1}; a_6, 4.9 mM^{-1}; w_l^0, 3×10^{-3} (mmHg·s)$^{-1}$.

Calibration parameters for pH titration curve. The ratio of enhancement factors $(E_R^{456.6} + 1)/(E_R^{456.6} + E_{RH}^{457} + 2)$ was obtained from experimental data at different irradiation powers (0.125, 0.25, 0.5, 1, 2 and 4 W), pH (in the range 6.4-7.4 and at points 5.0 and 10.0), contract agent (0.2, 0.4, 1 mM) and oxygen partial pressures (0, 7.6, 15.2, 38, 76 mmHg). The pH dependences of this ratio were analyzed by equation (11) using global fitting procedure, where pK was a fitting

parameter (pK=6.88±0.01) and R_{max} and R_{min} were found for each data set independently (see Fig. 2a, *Main Text*). The power dependence of parameters R_{max} and R_{min} was approximated using equations (13) yielding parameters s_1, s_2, s_3, s_4 equal to 30.7 μT^2, 32.7 μT^2, 4.7 μT^2, 38 μT^2 (see Fig. 2b, *Main Text*).

Calibration parameters for Pi measurements. The dependences of the normalized ratio $R_m = E_m^{456.8}/(E_R^{456.6} + E_{RH}^{457})$ on irradiation power (0.125, 0.25, 0.5, 1, 2, 4, 8 W) was obtained for solutions with phosphate buffer concentration 0, 1, 2, 4 mM at pH values from 6.4 to 7.4, pO_2 values 0, 15.2, 38, 76 mmHg and various contrast agent concentrations from 0.5 to 1.7 mM. The highest power level of 8 W was used for estimation of value $R_m^{max} = R_m(P \to \infty)$ and the enhancement factor dependences was smoothen using equation (22) to minimize calculation errors. Parameters b_0, b_1, b_2, b_3, b_4 were found using inorganic phosphate free solution data (totally 36 data sets) globally fitted by equation (14), (17) and (20) resulting values 1.20 μT, 0.95 μT/mM, -0.62 μT/mM, -0.015 μT/mmHg and 2.5. In order to calculate parameters b_5, b_6, b_7, b_8, b_9, b_{10} data sets for inorganic phosphate containing solutions (totally 52 data sets) was fitted using equations (14), (17), (20) and (21a) to obtain parameter g_{Pi}^0 for each data set. The dependence of g_{Pi}^0 function on pH was fitted by equation (21b) yielding parameter b_{10}=7.47 (see Figure S2a) The dependence of $h(C, pO_2, Pi)$ function on pO_2 value (see Figure S2b) and contrast agent concentration (see Figure S2c) were fitted using equation (21c) yielding values of parameters b_5=0.975 μT^2·mM^{-1}, b_6=0.075 μT^2·mmHg^{-1}·mM^{-1}, b_7=0.012 μT^2·mmHg^{-1}·mM^{-2}, b_8=5.75 μT^2·mM^{-2}, b_9=0.975 μT^2·mM^{-3}.

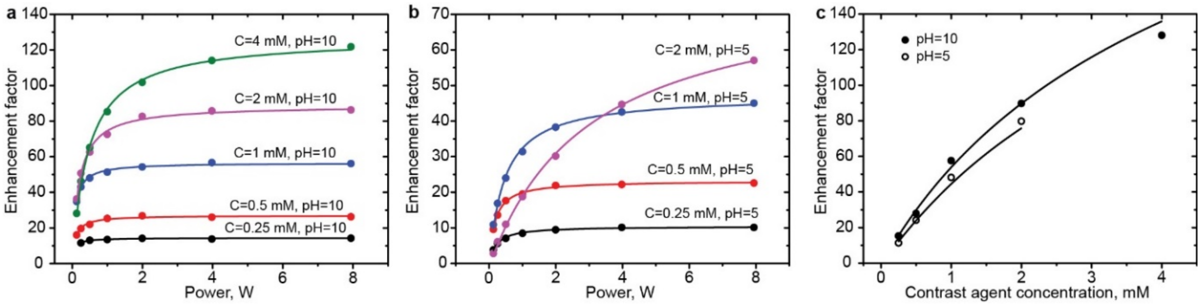

Figure S1. (**a**) The dependence of enhancement factor on irradiation power measured at different contrast agent concentrations in anaerobic conditions at pH 10. (**b**) The dependence of enhancement factor on irradiation power measured at different contrast agent concentrations in anaerobic conditions at pH 5. The solid lines are the best fits of equation (22) to the experimental data yielding the values of maximum enhancement. (**c**) The dependence of maximum enhancement on contrast agent concentration at pH values 5 and 10, the solid lines is the best fits of equation (10) to the maximum enhancement data yielding the values of coupling factors, ε_R and ε_{RH} , equal to 0.40±0.02 and 0.33±0.01 and enhancements at infinite power and contrast agent concentration, E_R^{inf} and E_{RH}^{inf} , equal to 270±4 and 221±5.

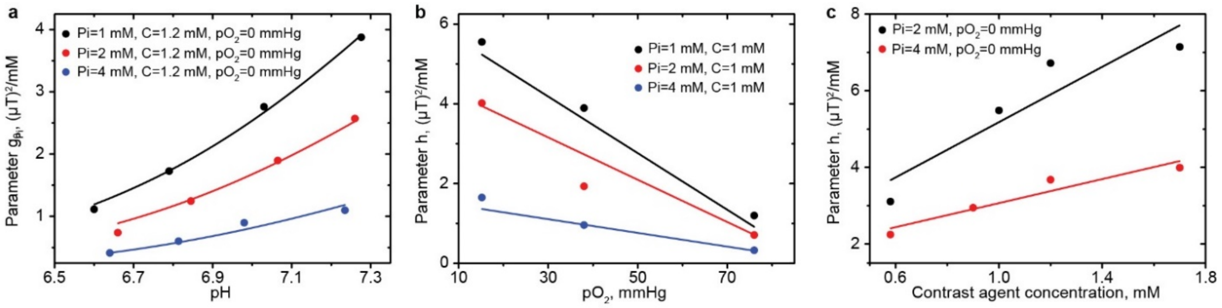

Figure S2. (**a**) The pH dependence of the parameter g_{Pi}^0 at different phosphate buffer concentrations (see equations (14), (17), (20) and (21a)) at C=1.2 mM, and pO_2=0 mmHg. The solid lines are the best fits of equation (21b). (**b**) The dependence of parameter h (see equation (21b)) on oxygen concentration at fixed values of Pi and C. The solid lines are the best fits of equation (21c). (**c**) The dependence of parameter h on C in anaerobic conditions at Pi values 2 and 4 mM (see equation (21b)). The solid lines are the best fits of equation (21c).

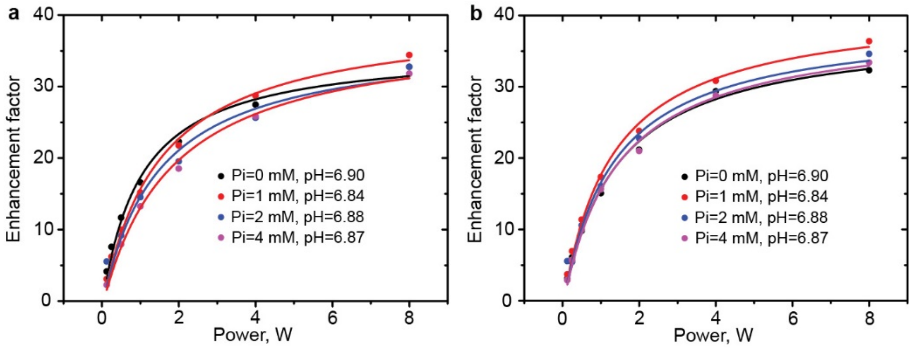

Figure. S3. The dependences of enhancement factors on irradiation power measured at spin probe concentration 1 mM, oxygen partial pressure 38 mmHg, pH≈6.9 and phosphate buffer concentrations (Pi) 0, 1, 2, 4 mM for deprotonated (**a**) and protonated (**b**) form of dpTAM. The solid lines are the best fits of equation (21) to the experimental data yielding the values of maximum enhancement laying in the range 39±2 and 40±2 for deprotonated and protonated forms.

prepared			calculated using deprotonated form data		calculated using protonated form data		calculated
C, mM	pO_2, mmHg	pH	C, mM	pO_2, mmHg	C, mM	pO_2, mmHg	pH
1	0	7.44	1.10 ± 0.03	0 ± 0	0.99 ± 0.06	0 ± 0	7.36 ± 0.05
1	0	7.26	1.04 ± 0.08	1 ± 3	1.03 ± 0.08	2 ± 2	7.22 ± 0.04
1	0	6.92	1.04 ± 0.04	0 ± 0	1.02 ± 0.04	0 ± 0	6.92 ± 0.03
1	0	6.63	1.07 ± 0.08	2 ± 2	1.06 ± 0.07	2 ± 2	6.66 ± 0.01
1	0	6.44	1.05 ± 0.08	1 ± 2	1.17 ± 0.07	3 ± 2	6.47 ± 0.02
1	0	6.92	1.04 ± 0.03	0 ± 0	1.02 ± 0.03	0 ± 0	6.92 ± 0.03
1	15.2	6.95	0.96 ± 0.08	17 ± 4	1.02 ± 0.08	17 ± 2	6.97 ± 0.01
1	38	6.89	1.08 ± 0.12	36 ± 5	1.03 ± 0.09	32 ± 4	6.93 ± 0.01
1	76	6.91	0.95 ± 0.21	75 ± 15	0.97 ± 0.18	68 ± 9	6.95 ± 0.02
1	0	6.92	1.00 ± 0.02	0 ± 0	1.05 ± 0.03	0 ± 0	6.90 ± 0.02
0.4	0	6.92	0.39 ± 0.01	0 ± 0	0.42 ± 0.02	0 ± 0	6.89 ± 0.02
0.2	0	6.91	0.17 ± 0.01	0 ± 0	0.17 ± 0.02	0 ± 0	6.83 ± 0.01

Table SI1. The set of solutions with premade C, pO_2 and pH values and corresponding calculated values for the deprotonated and protonated dpTAM form using eqns (2a) and (2b) and the fitting procedure described above for data presented in Figure 3 *Main Text*.

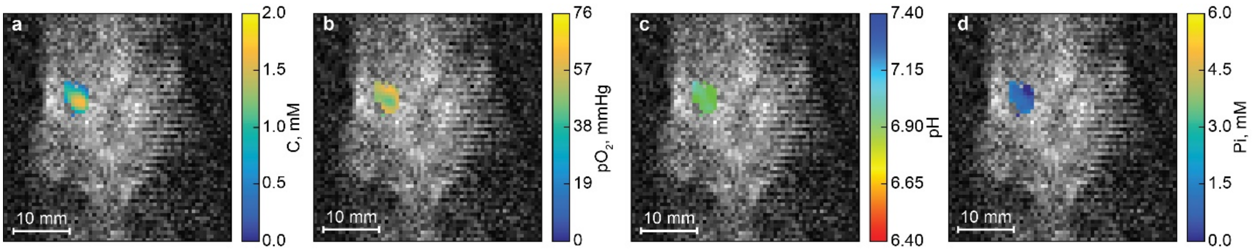

Figure S4. Multiparametric imaging of mouse mammary gland №1. (**a-d**) The contrast agent (C), pO_2, pH, and Pi maps. Acquisition parameters are: T_{EPR}, 500 ms; T_R, 700 ms; T_E, 37 ms; matrix, 64×64; field of view, 40×40 mm^2; slice thickness, 4 mm; total acquisition time, 4.3 min; imaging performed using powers 0.25 and 8 W and frequencies of EPR irradiation 456.6, 456.8 and 457 MHz; NMR frequency, 686.3 kHz.

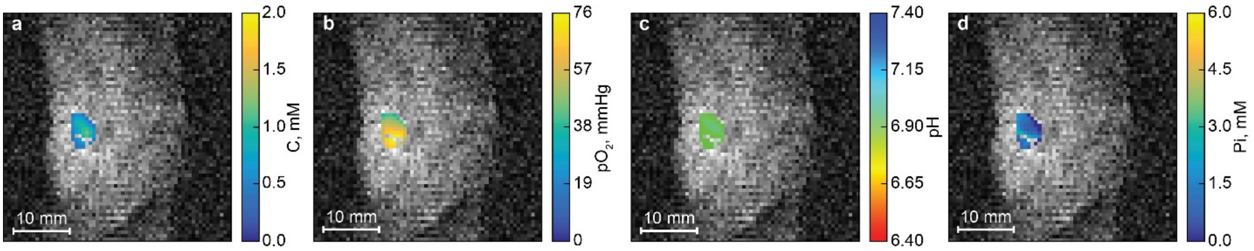

Figure S5. Multiparametric imaging of mouse mammary gland №2. (**a-d**) The contrast agent (C), pO_2, pH, and Pi maps. Acquisition parameters are: T_{EPR}, 500 ms; T_R, 700 ms; T_E, 37 ms; matrix, 64×64; field of view, 40×40 mm^2; slice thickness, 4 mm; total acquisition time, 4.3 min; imaging performed using powers 0.5 and 8 W and frequencies of EPR irradiation 456.6, 456.8 and 457 MHz; NMR frequency, 686.3 kHz.

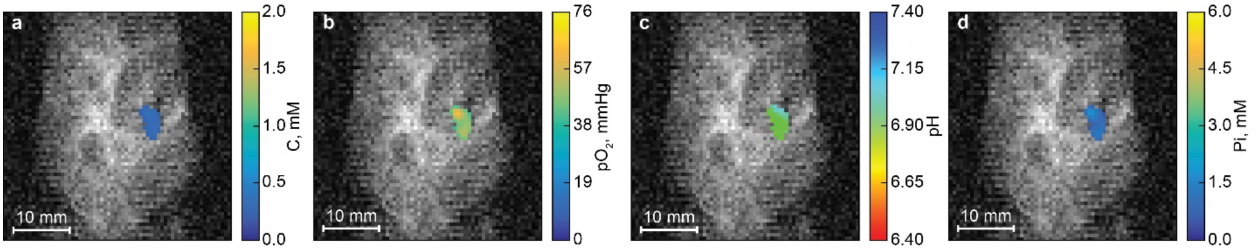

Figure S6. Multiparametric imaging of mouse mammary gland №3. (**a-d**) The contrast agent (C), pO_2, pH, and Pi maps. Acquisition parameters are: T_{EPR}, 500 ms; T_R, 700 ms; T_E, 37 ms; matrix, 64×64; field of view, 40×40 mm^2; slice thickness, 4 mm; total acquisition time, 4.3 min; imaging performed using powers 0.5 and 8 W and frequencies of EPR irradiation 456.6, 456.8 and 457 MHz; NMR frequency, 686.3 kHz.

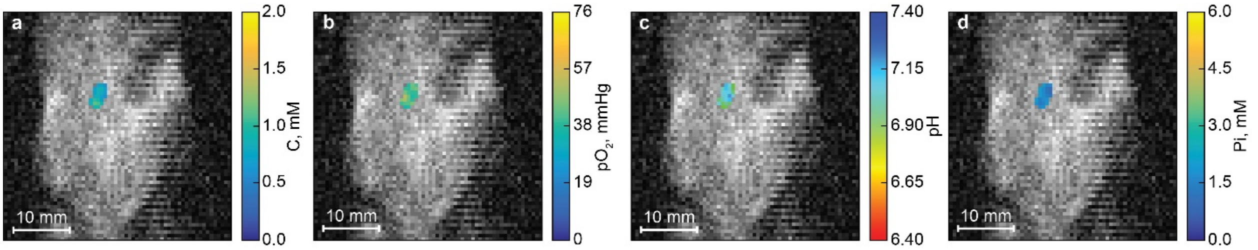

Figure S7. Multiparametric imaging of mouse mammary gland №4. (**a-d**) The contrast agent (C), pO_2, pH, and Pi maps. Acquisition parameters are: T_{EPR}, 500 ms; T_R, 700 ms; T_E, 37 ms; matrix, 64×64; field of view, 40×40 mm^2; slice thickness, 4 mm; total acquisition time, 4.3 min; imaging performed using powers 0.25 and 8 W and frequencies of EPR irradiation 456.6, 456.8 and 457 MHz; NMR frequency, 686.3 kHz.

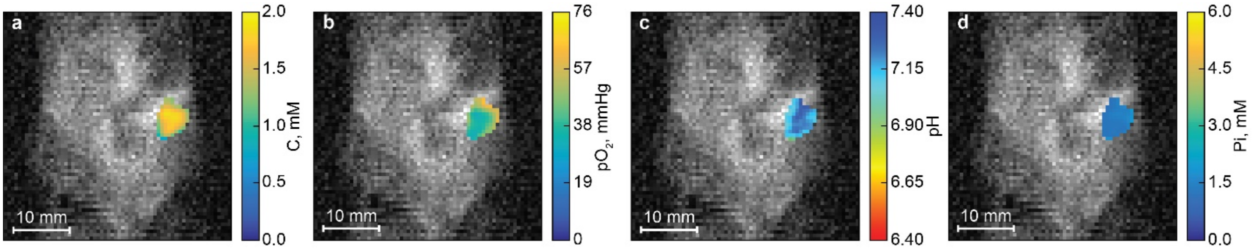

Figure S8. Multiparametric imaging of mouse mammary gland №5. (**a-d**) The contrast agent (C), pO_2, pH, and Pi maps. Acquisition parameters are: T_{EPR}, 500 ms; T_R, 700 ms; T_E, 37 ms; matrix, 64×64; field of view, 40×40 mm^2; slice thickness, 4 mm; total acquisition time, 4.3 min; imaging performed using powers 0.5 and 8 W and frequencies of EPR irradiation 456.6, 456.8 and 457 MHz; NMR frequency, 686.3 kHz.

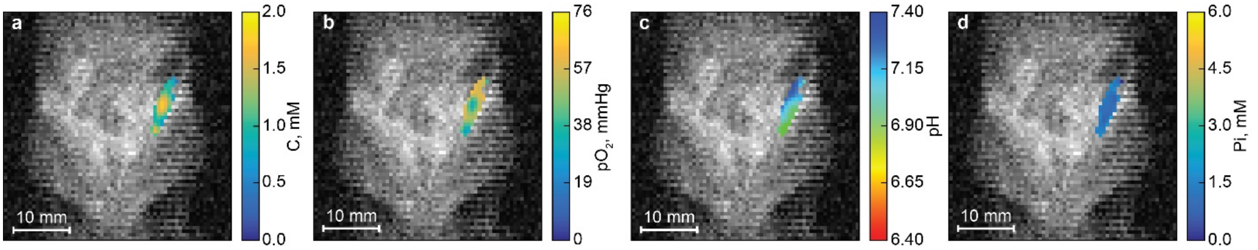

Figure S9. Multiparametric imaging of mouse mammary gland №6. (**a-d**) The contrast agent (C), pO_2, pH, and Pi maps. Acquisition parameters are: T_{EPR}, 500 ms; T_R, 700 ms; T_E, 37 ms; matrix, 64×64; field of view, 40×40 mm^2; slice thickness, 4 mm; total acquisition time, 4.3 min; imaging performed using powers 0.25 and 8 W and frequencies of EPR irradiation 456.6, 456.8 and 457 MHz; NMR frequency, 686.3 kHz.

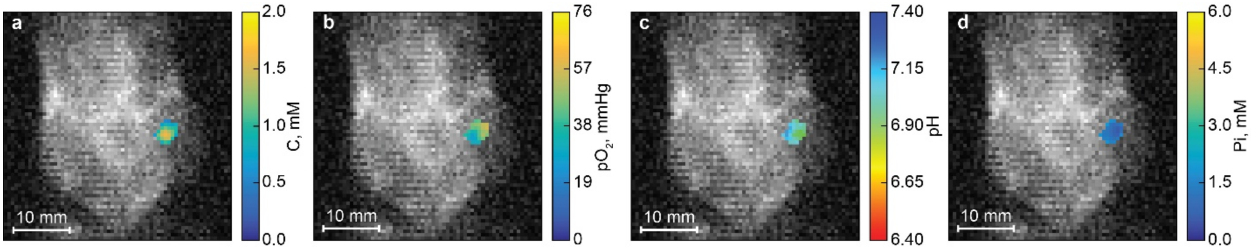

Figure S10. Multiparametric imaging of mouse mammary gland №7. (**a-d**) The contrast agent (C), pO_2, pH, and Pi maps. Acquisition parameters are: T_{EPR}, 500 ms; T_R, 700 ms; T_E, 37 ms; matrix, 64×64; field of view, 40×40 mm^2; slice thickness, 4 mm; total acquisition time, 4.3 min; imaging performed using powers 0.25 and 8 W and frequencies of EPR irradiation 456.6, 456.8 and 457 MHz; NMR frequency, 686.3 kHz.

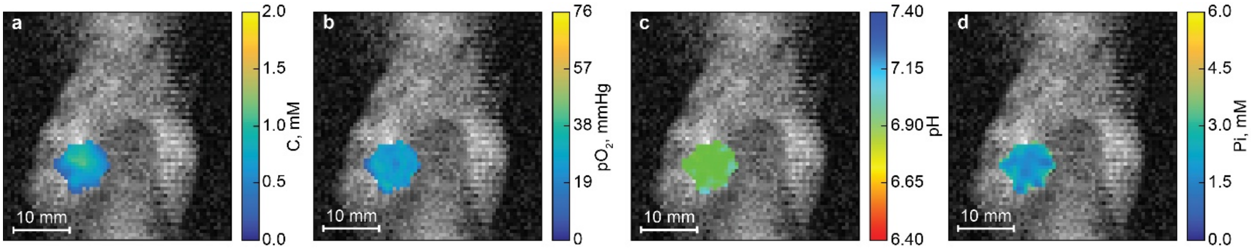

Figure S11. Multiparametric imaging of mouse tumor №1. (**a-d**) The contrast agent (C), pO_2, pH, and Pi maps. Tumor volume of 1.4 cm^3. Acquisition parameters are: T_{EPR}, 500 ms; T_R, 700 ms; T_E, 37 ms; matrix, 64×64; field of view, 40×40 mm^2; slice thickness, 4 mm; total acquisition time, 4.3 min; imaging performed using powers 0.25 and 8 W and frequencies of EPR irradiation 456.6, 456.8 and 457 MHz; NMR frequency, 686.3 kHz.

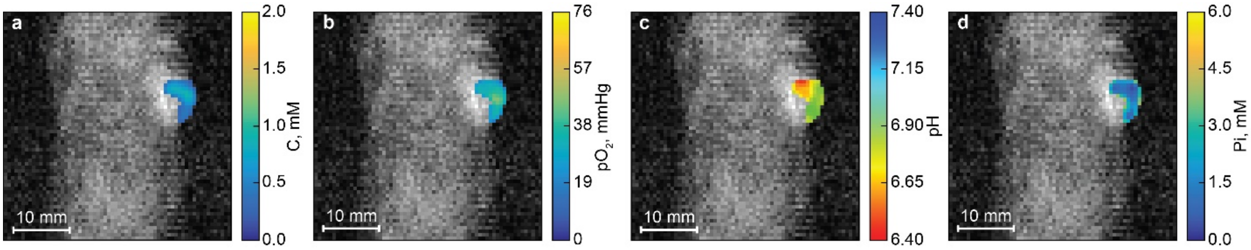

Figure S12. Multiparametric imaging of mouse tumor №2. (**a-d**) The contrast agent (C), pO_2, pH, and Pi maps. Tumor volume of 0.2 cm^3. Acquisition parameters are: T_{EPR}, 500 ms; T_R, 700 ms; T_E, 37 ms; matrix, 64×64; field of view, 40×40 mm^2; slice thickness, 4 mm; total acquisition time, 4.3 min; imaging performed using powers 0.25 and 8 W and frequencies of EPR irradiation 456.6, 456.8 and 457 MHz; NMR frequency, 686.3 kHz.

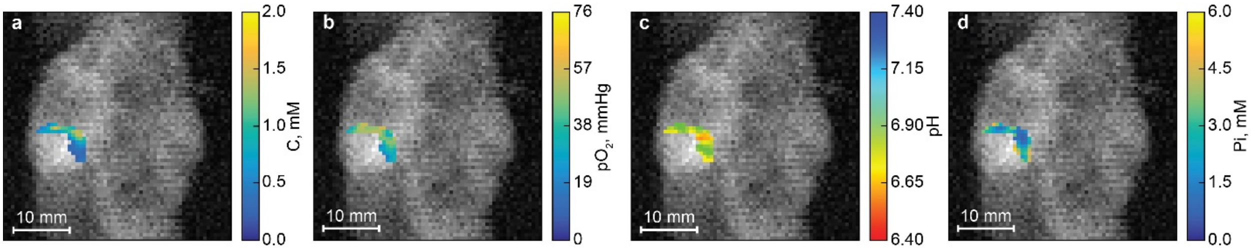

Figure S13. Multiparametric imaging of mouse tumor №3. (**a-d**) The contrast agent (C), pO_2, pH, and Pi maps. Tumor volume of 0.4 cm^3. Acquisition parameters are: T_{EPR}, 500 ms; T_R, 700 ms; T_E, 37 ms; matrix, 64×64; field of view, 40×40 mm^2; slice thickness, 4 mm; total acquisition time, 4.3 min; imaging performed using powers 0.25 and 8 W and frequencies of EPR irradiation 456.6, 456.8 and 457 MHz; NMR frequency, 686.3 kHz.

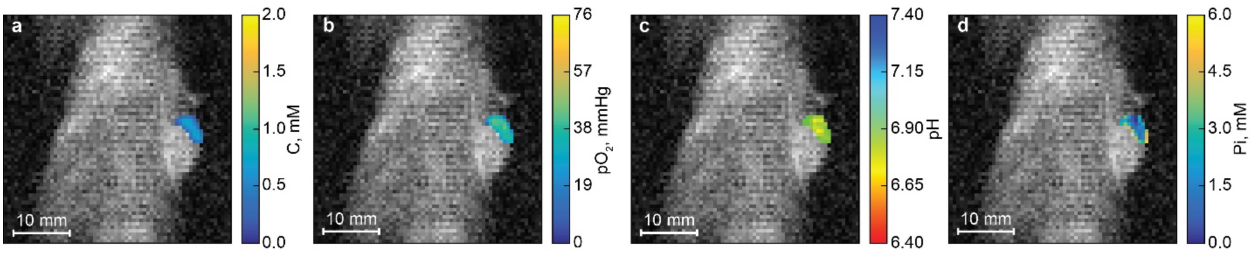

Figure S14. Multiparametric imaging of mouse tumor №4. (**a-d**) The contrast agent (C), pO_2, pH, and Pi maps. Tumor volume 0.25 cm^3. Acquisition parameters are: T_{EPR}, 500 ms; T_R, 700 ms; T_E, 37 ms; matrix, 64×64; field of view, 40×40 mm^2; slice thickness, 4 mm; total acquisition time, 4.3 min; imaging performed using powers 0.25 and 8 W and frequencies of EPR irradiation 456.6, 456.8 and 457 MHz; NMR frequency, 686.3 kHz.

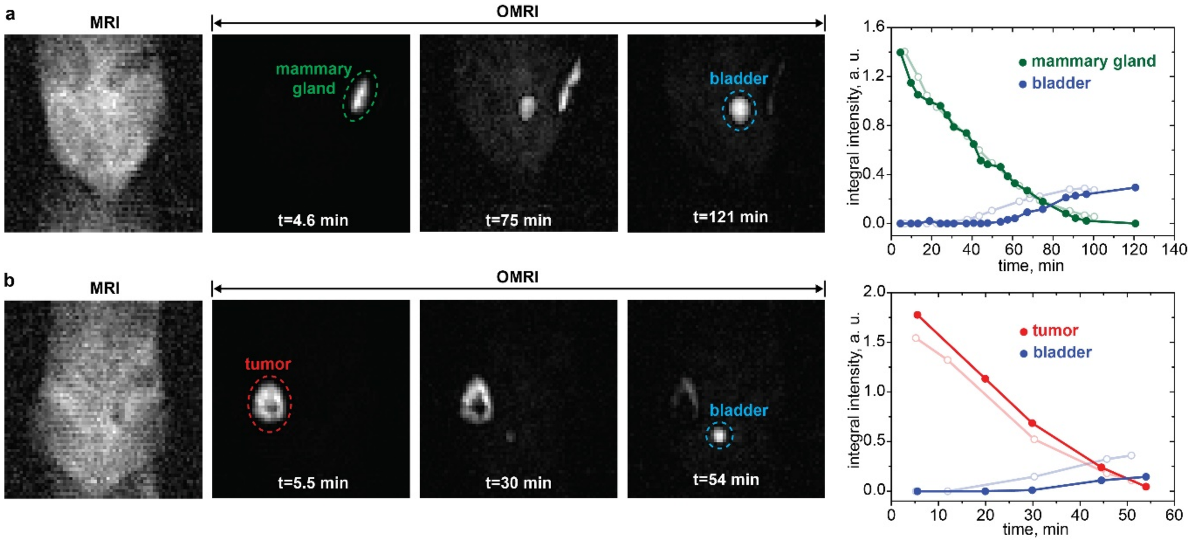

Figure S15. Longitudinal imaging of healthy (a) and tumor-bearing (b) mouse injected with the dpTAM probe. The anatomical MRI, OMRI images (recorded at different time after dpTAM injection), as well as the kinetics of signal intensity in both tumor/mammary gland and bladder, are shown for two separate experiments performed in different animals (shown in filled and empty symbols, correspondingly). The characteristic time of dpTAM wash out from tumor, and the mammary gland was found to be equal to 26 min and 43 min, correspondingly. The characteristic time of dpTAM signal accumulation in the bladder was found to be equal 34-40 min and 58-78 min for tumor-bearing and healthy mouse, correspondingly. Acquisition parameters are: T_{EPR}, 500 ms; T_R, 700 ms; T_E, 37 ms; matrix, 64×64; field of view, 40×40 mm^2; slice thickness, 100 mm; acquisition time for one OMRI image, 24 s; power and frequency of EPR irradiation, 8 W and 456.6 MHz; NMR frequency, 686.3 kHz.

Mouse type	C, mM	pO_2, mmHg	pH	Pi, mM	Tumor volume, cm^3
Mammary gland № 1	1.0 ± 0.4	53 ± 6	7.00 ± 0.06	0.9 ± 0.4	NA
Mammary gland № 2	0.7 ± 0.2	58 ± 11	6.98 ± 0.04	1.0 ± 0.7	NA
Mammary gland № 3	0.30 ± 0.03	49 ± 6	7.00 ± 0.07	1.1 ± 0.3	NA
Mammary gland № 4	0.8 ± 0.1	43 ± 4	7.1 ± 0.1	1.6 ± 0.3	NA
Mammary gland № 5	1.5 ± 0.2	45 ± 9	7.20 ± 0.06	1.4 ± 0.2	NA
Mammary gland № 6	1.2 ± 0.3	50 ± 9	7.1 ± 0.1	1.2 ± 0.3	NA
Mammary gland № 7	1.0 ± 0.3	42 ± 9	7.10 ± 0.07	1.2 ± 0.3	NA
Tumor № 1	0.7 ± 0.2	26 ± 5	6.95 ± 0.07	2.2 ± 0.6	1.4
Tumor № 2	0.6 ± 0.2	32 ± 7	6.7 ± 0.1	1.8 ± 0.9	0.2
Tumor № 3	0.7 ± 0.3	41 ± 10	6.7 ± 0.1	2.6 ± 1.2	0.4
Tumor № 4	0.5 ± 0.2	33 ± 9	6.78 ± 0.07	2.2 ± 1.4	0.25

Table SI2. The mean contrast agent (C), pO_2, pH, Pi values, and their standard deviations for imaging data presented on Figures S4-S14. The designation NA means not available.

References

1 K. H. Hausser , D. S. Dynamic Nuclear Polarization in Liquids *Advances in Magnetic Resonance*, 79 (1968).

2 Gorodetskii, A. A. *et al.* Oxygen-induced leakage of spin polarization in Overhauser-enhanced magnetic resonance imaging: Application for oximetry in tumors. *J Magn Reson* **297**, 42-50, doi:10.1016/j.jmr.2018.10.005 (2018).

3 Armstrong, B. D. & Han, S. A new model for Overhauser enhanced nuclear magnetic resonance using nitroxide radicals. *J Chem Phys* **127**, 104508, doi:10.1063/1.2770465 (2007).

4 Golman, K. *et al.* Dynamic in vivo oxymetry using overhauser enhanced MR imaging. *J Magn Reson Imaging* **12**, 929-938 (2000).

5 Bobko, A. A., Dhimitruka, I., Zweier, J. L. & Khramtsov, V. V. Fourier transform EPR spectroscopy of trityl radicals for multifunctional assessment of chemical microenvironment. *Angew Chem Int Ed Engl* **53**, 2735-2738, doi:10.1002/anie.201310841 (2014).

6 Thacker, J., Zhang, J. L., Franklin, T. & Prasad, P. BOLD quantified renal pO2 is sensitive to pharmacological challenges in rats. *Magn Reson Med* **78**, 297-302, doi:10.1002/mrm.26367 (2017).

7 Ardenkjaer-Larsen, J. H. *et al.* EPR and DNP properties of certain novel single electron contrast agents intended for oximetric imaging. *Journal of Magnetic Resonance* **133**, 1-12, doi:DOI 10.1006/jmre.1998.1438 (1998).
